# bayNorm: Bayesian gene expression recovery, imputation and normalization for single-cell RNA-sequencing data

**DOI:** 10.1093/bioinformatics/btz726

**Published:** 2019-10-04

**Authors:** Wenhao Tang, François Bertaux, Philipp Thomas, Claire Stefanelli, Malika Saint, Samuel Marguerat, Vahid Shahrezaei

**Affiliations:** 1 Department of Mathematics, Faculty of Natural Sciences, Imperial College, London SW7 2AZ, UK; 2 MRC London Institute of Medical Sciences (LMS), London W12 0NN, UK; 3 Faculty of Medicine, Institute of Clinical Sciences (ICS), Imperial College London, London W12 0NN, UK

## Abstract

**Motivation:**

Normalization of single-cell RNA-sequencing (scRNA-seq) data is a prerequisite to their interpretation. The marked technical variability, high amounts of missing observations and batch effect typical of scRNA-seq datasets make this task particularly challenging. There is a need for an efficient and unified approach for normalization, imputation and batch effect correction.

**Results:**

Here, we introduce bayNorm, a novel Bayesian approach for scaling and inference of scRNA-seq counts. The method’s likelihood function follows a binomial model of mRNA capture, while priors are estimated from expression values across cells using an empirical Bayes approach. We first validate our assumptions by showing this model can reproduce different statistics observed in real scRNA-seq data. We demonstrate using publicly available scRNA-seq datasets and simulated expression data that bayNorm allows robust imputation of missing values generating realistic transcript distributions that match single molecule fluorescence *in situ* hybridization measurements. Moreover, by using priors informed by dataset structures, bayNorm improves accuracy and sensitivity of differential expression analysis and reduces batch effect compared with other existing methods. Altogether, bayNorm provides an efficient, integrated solution for global scaling normalization, imputation and true count recovery of gene expression measurements from scRNA-seq data.

**Availability and implementation:**

The R package ‘bayNorm’ is publishd on bioconductor at https://bioconductor.org/packages/release/bioc/html/bayNorm.html. The code for analyzing data in this article is available at https://github.com/WT215/bayNorm_papercode.

**Supplementary information:**

Supplementary data are available at *Bioinformatics* online.

## 1 Introduction

Single-cell RNA-sequencing (scRNA-seq) is a method of choice for profiling global gene expression heterogeneity across tissues in health and disease (Baslan and Hicks, 2017; Chen *et al.*, 2018). Because it relies on the detection of minute amounts of biological material, namely the RNA content of one single cell, scRNA-seq is characterized by unique and strong technical biases. These arise mainly because scRNA-seq library preparation protocols recover only a small fraction of the total RNA molecules present in each cell. As a result, scRNA-seq data are usually very sparse with many genes showing missing values (i.e. zero values, also called dropouts). The fraction of all transcripts recovered from a cell is called capture efficiency and varies from cell to cell, resulting in strong technical variability in transcripts expression levels and dropouts rates. Moreover, capture efficiencies tend to vary between experimental batches resulting in confounding ‘batch effects’. Correcting for these biases in order to recover scRNA-seq counts reflecting accurately the original numbers of transcripts present in a cell remains a major challenge in the field (Bacher and Kendziorski, 2016; Vallejos *et al.*, 2017; Ziegenhain *et al.*, 2018).

A common approach to scRNA-seq normalization is the use of cell-specific global scaling factors. These methods are based on principles developed for normalization of bulk RNA-seq experiments and assume that gene-specific biases are small (Vallejos *et al.*, 2017). Typically, read counts per cell are divided by a cell-specific scaling factor estimated either from spike-in controls (Brennecke *et al.*, 2013), or directly from the transcriptome data using methods developed initially for bulk RNA-seq (Love *et al.*, 2014; Robinson and Oshlack, 2010; Robinson and Smyth, 2007) or specifically for scRNA-seq (Lun *et al.*, 2016; Vallejos *et al.*, 2015). A recent method called SCnorm extended the global scaling approach by introducing different scaling factors for different expression groups (Bacher *et al.*, 2017).

Importantly, scaling methods do not correct for cell-to-cell variations in dropout rates, as genes with zero counts remain zero after division by a scaling factor. Several approaches have been designed to tackle this problem. A series of methods use zero-inflated distribution functions, to explicitly model the dropout characteristics (Finak *et al.*, 2015; Kharchenko *et al.*, 2014; Pierson and Yau, 2015). Alternatively, other studies have proposed to infer dropouts based on expression values pooled across cells or genes (Eraslan *et al.*, 2019; Huang *et al.*, 2018; Li and Li, 2018; van Dijk *et al.*, 2018). For instance, scImpute pools expression values across similar cell subpopulations in each dataset and imputes dropouts using a Gamma-Normal mixture model and population-specific thresholds (Li and Li, 2018). Similarly, the MAGIC package is based on pooling gene expression values across cells using a network-based similarity metric (van Dijk *et al.*, 2018). Another method is based on K-nearest neighbor smoothing, which uses Poisson distribution and aggregate information from similar cells (Wagner *et al.*, 2018). Conversely, the SAVER approach pools expression values across genes within each cell using a Gamma-Poisson Bayesian model (Huang *et al.*, 2018). The Gamma-Poisson model is also used in two other packages called Splatter and scVI for simulating and normalizing scRNA-seq data, respectively (Lopez *et al.*, 2018; Zappia *et al.*, 2017). scVI belongs to new class of approaches which implement deep learning methods (Ding *et al.*, 2018; Eraslan *et al.*, 2019; Grønbech *et al.*, 2018; Lopez *et al.*, 2018; Wang and Gu, 2018). For instance, DCA, an autoencoder method, utilizes a zero-inflated negative binomial noise model (Eraslan *et al.*, 2019). Apart from Gamma-Poisson model, multivariate Normal distribution was assumed for the log transformed data in BISCUIT, which is a Bayesian method that uses an iterative approach to normalization and clustering Azizi *et al.* (2018) and Prabhakaran *et al.* (2016). However, the log transformation can affect downstream analysis (see Lun, 2018; for more discussion about issues in log transformation of scRNA-seq data). Experimental batch-to-batch variations are another common source of technical variability in scRNA-seq data. The origin of batch effects is not fully understood but results at least in part from differences in average capture efficiencies across experiments (Hicks *et al.*, 2018). Recently, several methods have been specifically developed to remove batch effect in scRNA-seq data (Butler *et al.*, 2018; Haghverdi *et al.*, 2018; Kiselev *et al.*, 2018).

Many of the methods discussed above treat normalization, imputation and batch effect correction as separate tasks. Moreover, some methods rely on strong assumptions such as various zero-inflation models. Here we provide a detailed account of a novel integrated approach called bayNorm, which performs all the processing steps discussed above at the same time using minimal assumptions. We compared its performance with a series of available packages focusing on true count recovery, differential expression (DE) analysis and batch effect correction.

## 2 Materials and methods

**Fig. 1.**
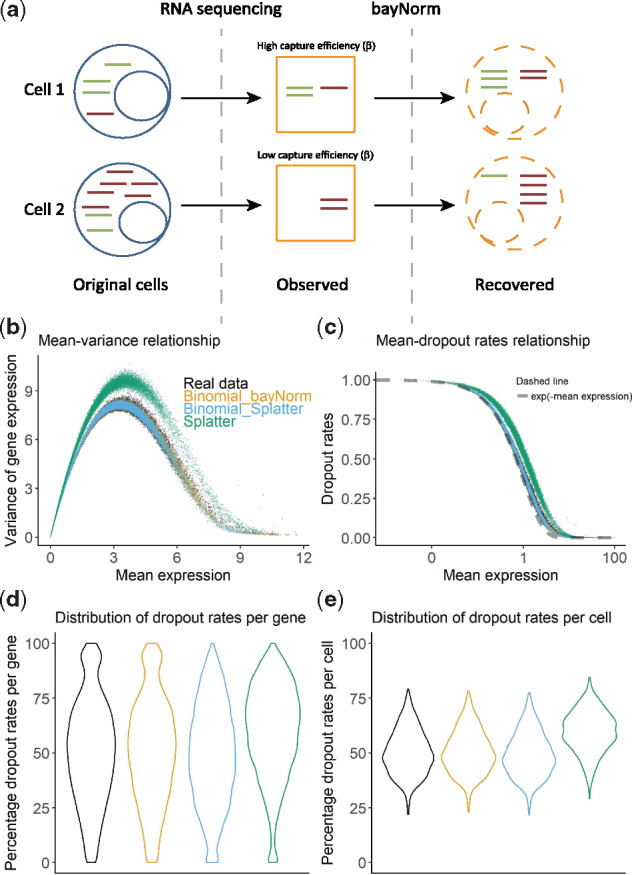
A binomial model of mRNA capture is consistent with the statistics of raw experimental scRNA-seq data. (**a**) Cartoon illustration of the bayNorm approach. Only a fraction of the total number of mRNAs present in the cell is captured during scRNA-seq library preparation. This occurs with a global probability called capture efficiency (*β*). Using cell-specific estimates of *β*, bayNorm aims at recovering the original number of mRNA of each gene present in each cell. Comparisons between raw experimental scRNA-seq data from the Klein study (Klein *et al.*, 2015) and synthetic data obtained using the Binomial_bayNorm (orange), Binomial_Splatter (blue) or Splatter (Zappia *et al.*, 2017) (green) simulation protocols (see Supplementary Note S2 for details). (**b**) Variance versus mean expression relationship. (**c**) Dropout rates versus mean expression relationship (note that Binomial_Splatter and Binomial_bayNorm are on top of each other in this panel). The dotted line shows the $e^{\left(-\text{Mean}\;\text{expression}\right)}$ function. (**d**) Distribution of dropout values per gene. (**e**) Distribution of dropout values per cell. (Color version of this figure is available at *Bioinformatics* online.)

A scRNA-seq dataset is typically represented in a matrix of dimension *P* × *Q*, where *P* denotes the total number of genes observed and *Q* denotes the total number of cells studied. The element *x_ij_* (i∈{1,2,…,P} and j∈{1,2,…,Q}) in the matrix represents the number of transcripts reported for the *i*th gene in the *j*th cell. This is equal to the total number of sequencing reads mapping to that gene in that cell for a non-unique molecular identifier (UMI) protocol. For UMI-based protocols this is equal to the number of individual UMIs mapping to each gene (Parekh *et al.*, 2018; Smith *et al.*, 2017). The matrix can include data from different groups or batches of cells, representing different biological conditions. This can be represented as a vector of labels for the cell groups or conditions (*C_j_*). bayNorm generates for each gene (*i*) in each cell (*j*) a posterior distribution of original expression counts ($x_{\mathrm{ij}}^{0}$), given the observed scRNA-seq read count for that gene (*x_ij_*) (Fig. 1a).

A common approach for normalizing scRNA-seq data is based on the use of a global scaling factor (*s_j_*), ignoring any gene-specific biases (for a recent review see Vallejos *et al.*, 2017). The normalized data ${\tilde{x}}_{\mathrm{ij}}$ is obtained by dividing the raw data for each cell *j* by its global scaling factor *s_j_*:

(1) $${\tilde{x}}_{\mathrm{ij}}=\frac{x_{\mathrm{ij}}}{s_{j}}$$

In bayNorm, we implement global scaling using a Bayesian approach to infer the original transcript counts in each cell. We assume given the original number of transcripts in the cell ($x_{\mathrm{ij}}^{0}$), the number of transcripts observed (*x_ij_*) follows a Binomial model with probability *β_j_* (Klein *et al.*, 2015), which we refer to as capture efficiency and it represents the probability of original transcripts in the cell to be observed for a cell with average size (or average transcript content). The capture efficiencies are proportional to global scaling factors normalized by an estimate of mean capture efficiency $\bar{\beta }$ (the average fraction of original transcripts that are observed across all cells) for the experiment and correct for cell-to-cell variation in transcript capture and original transcript content (see Supplementary Note S1). In addition, we assume that the original number or true count of the *i*th gene in the *j*th cell ($x_{\mathrm{ij}}^{0}$) follows Negative Binomial distribution with parameters mean (*μ*) and size (or dispersion parameter, *ϕ*), such that:

$$\mathrm{Pr}(x_{\mathrm{ij}}^{0}=n|\phi_{i},\mu_{i})=\frac{\Gamma (n+\phi_{i})}{\Gamma (\phi_{i})n!}{\left(\frac{\phi_{i}}{\mu_{i}+\phi_{i}}\right)}^{\phi_{i}}{\left(\frac{\mu_{i}}{\mu_{i}+\phi_{i}}\right)}^{n}.$$

So, overall we have the following model:

(2) $$\begin{array}{cc} & x_{\mathrm{ij}}|x_{\mathrm{ij}}^{0}∼\text{Binom}(x_{\mathrm{ij}}^{0},\text{prob}=\beta_{j}),\\ & x_{\mathrm{ij}}^{0}∼\text{NB}(\text{mean}=\mu_{i},\text{size}=\phi_{i}). \end{array}$$

Using the Bayes rule, we have the following posterior distribution of original number of mRNAs for each gene in each cell:

(3) $$\underset{\text{Posterior}}{\underset{︸}{\mathrm{Pr}(x_{ij}^{0}|x_{ij},\beta_{j},\mu_{i},\phi_{i})}}=\frac{\overset{\text{Likelihood}}{\overset{︷}{\mathrm{Pr}(x_{ij}|x_{ij}^{0},\beta_{j})}}\times \overset{\text{Prior}}{\overset{︷}{\mathrm{Pr}(x_{ij}^{0}|\mu_{i},\phi_{i})}}}{\underset{\text{Marginal likelihood}}{\underset{︸}{\mathrm{Pr}(x_{ij}|\mu_{i},\phi_{i},\beta_{j})}}}$$

The prior parameters *μ* and *ϕ* of each gene were estimated using an empirical Bayesian method by pooling information across cells as discussed in detail in Supplementary Note S1. The estimation is termed ‘global’, if priors informed by combining all cells in the study regardless of their conditions or batch (*C_j_*) and is termed ‘local’, if the prior is estimated by pooling information across specific cell groups (*C_j_*).

The marginal likelihood for gene *i* in cell *j* is

(4) $$\begin{array}{c} \mathrm{Pr}(x_{\mathrm{ij}}|\mu_{i},\phi_{i},\beta_{j})=\sum_{n=0}^{+\infty }\underset{\text{Binomial}}{\underset{︸}{(\begin{array}{c} n\\ x_{\mathrm{ij}} \end{array})\beta_{j}^{x_{ij}}{\left(1-\beta_{j}\right)}^{n-x_{\mathrm{ij}}}}}\\ \times \underset{\text{Negative Binomial}}{\underset{︸}{\left(\begin{array}{c} n+\phi_{i}-1\\ \phi_{i}-1 \end{array}\right){\left(\frac{\phi_{i}}{\mu_{i}+\phi_{i}}\right)}^{\phi_{i}}{\left(\frac{\mu_{i}}{\mu_{i}+\phi_{i}}\right)}^{n}}}\\ =\underset{\text{Negative Binomial}}{\underset{︸}{\left(\begin{array}{c} x_{\mathrm{ij}}+\phi_{i}-1\\ \phi_{i}-1 \end{array}\right){\left(\frac{\phi_{i}}{\mu_{i}\beta_{j}+\phi_{i}}\right)}^{\phi_{i}}{\left(\frac{\mu_{i}\beta_{j}}{\mu_{i}\beta_{j}+\phi_{i}}\right)}^{x_{\mathrm{ij}}}}}, \end{array}$$

which follows from using

(5) $$\left(\begin{array}{c} n+\phi_{i}-1\\ \phi_{i}-1 \end{array}\right)\left(\begin{array}{c} n\\ x_{\mathrm{ij}} \end{array}\right)=\left(\begin{array}{c} x_{\mathrm{ij}}+\phi_{i}-1\\ \phi_{i}-1 \end{array}\right)\left(\begin{array}{c} n+\phi_{i}-1\\ n-x_{\mathrm{ij}} \end{array}\right),$$

and

(6) $$\begin{array}{c} \sum_{n=x_{\mathrm{ij}}}^{+\infty }z^{n}\left(\begin{array}{c} \phi_{i}+n-1\\ n-x_{\mathrm{ij}} \end{array}\right)=\sum_{m=0}^{+\infty }z^{m+x_{\mathrm{ij}}}\left(\begin{array}{c} \phi_{i}+m+x_{\mathrm{ij}}-1\\ m \end{array}\right)\\ =\frac{z^{x_{\mathrm{ij}}}}{{\left(1-z\right)}^{\phi_{i}+x_{\mathrm{ij}}}}, \end{array}$$

with $z=\frac{\mu_{i}}{\mu_{i}+\phi_{i}}(1-\beta_{j})$ in Equation (4). Hence we have that the number of transcripts reported for the *i*th gene in the *j*th cell

(7) $$x_{\mathrm{ij}}∼\text{NB}(\text{mean}=\mu_{i}\beta_{j},\text{size}=\phi_{i}),$$

has a Negative Binomial distribution with mean $\mu_{i}\beta_{j}$ and size *ϕ_i_*.

It can also be shown that the posterior distribution of $x_{\mathrm{ij}}^{0}$ is a shifted Negative Binomial distribution. To sample from the posterior distribution, we note that the original count can be expressed as

(8) $$x_{\mathrm{ij}}^{0}=x_{\mathrm{ij}}+\zeta_{\mathrm{ij}},$$

where *ζ_ij_* is the *lost* count satisfying

(9) $$\zeta_{\mathrm{ij}}∼\text{NB}(\text{mean}=\frac{\mu_{i}(1-\beta_{j})(x_{\mathrm{ij}}+\phi_{i})}{\mu_{i}\beta_{j}+\phi_{i}},\text{size}=x_{\mathrm{ij}}+\phi_{i}).$$

The posterior mean and variance then evaluate to

(10) $$E[x_{\mathrm{ij}}^{0}]=x_{\mathrm{ij}}\frac{\mu_{i}+\phi_{i}}{\mu_{i}\beta_{j}+\phi_{i}}+\mu_{i}\frac{\phi_{i}-\phi_{i}\beta_{j}}{\mu_{i}\beta_{j}+\phi_{i}},$$

(11) $$\text{Var}[x_{\mathrm{ij}}^{0}]=\frac{\left(x_{\mathrm{ij}}+\phi_{i})\mu_{i}(1-\beta_{j})(\mu_{i}+\phi_{i}\right)}{{\left(\phi_{i}+\mu_{i}\beta_{j}\right)}^{2}}.$$

Note that when *ϕ_i_* is small, the mean of posterior tends to $\frac{x_{\mathrm{ij}}}{\beta_{j}}$. After estimating the posterior distribution for each gene in each cell, we can either sample a certain number of draws from it (3D array output, see Supplementary Fig. S1) or extract the mean or maximum a posteriori probability (MAP; Gelman *et al.*, 2014; 2D array output, see Supplementary Fig. S1). More details on the use of Binomial distribution and estimation of *β* and priors can be found in the Supplementary Note S1 and pseudo code (Algorithm 1) in the Supplementary Note.

## 3 Results

### 3.1 The bayNorm model reproduces statistics of real scRNA-seq data

bayNorm models the true transcript counts in each cell using a Bayesian approach to global scaling normalization. The two original aspects of the method are: (i) the use of the binomial likelihood function, and (ii) the use of shrinkage methods to estimate prior parameters (*μ* and *ϕ*). The bayNorm likelihood function $\mathrm{Pr}(x_{\mathrm{ij}}|x_{\mathrm{ij}}^{0},\beta_{j})$ is assumed to be binomial as it describes the random sampling of a fraction of a cell transcriptome with constant probability. This is a simple model of transcript capture in scRNA-seq (Klein *et al.*, 2015) and we therefore hypothesized that it would be a good choice for the bayNorm likelihood function. For the prior $\mathrm{Pr}(x_{\mathrm{ij}}^{0})$, we assume a negative binomial model, which describes the bursty distribution of mRNAs in simple models of gene expression (Raj *et al.*, 2006; Shahrezaei and Swain, 2008) and is also commonly used in RNA-seq analysis Love *et al.* (2014). Gene-specific prior parameters are estimated using an empirical Bayes approach by pooling gene expression values across multiple cells (see Supplementary Note S1 and Supplementary Figs S27 and S28 for details). Although shrinkage methods have been commonly used to estimate dispersion (see e.g. Love *et al.*, 2014; Robinson and Smyth, 2007), the use of empirical Bayes shrinkage approaches for estimation of the mean is less common (but see Huang *et al.*, 2018; Love *et al.*, 2014; Zhu *et al.*, 2018). bayNorm normalized count of gene *i* in cell *j* is either a point estimate from posterior (mean/MAP) (2D array output) or samples of the corresponding posterior distribution (3D array output). The bayNorm 2D or 3D output can be used for further downstream analysis.

To validate our choice of binomial likelihood model and prior estimates, we generated simulated scRNA-seq data based on these assumptions and investigated how closely they captured statistics of several published scRNA-seq datasets (Fig. 1b–e and Supplementary Figs S2–S7; Bacher *et al.*, 2017; Klein *et al.*, 2015; Torre *et al.*, 2018; Tung *et al.*, 2017). The simulations assumed that mRNA counts per cell followed negative binomial distributions and used gene-specific priors obtained with bayNorm (Fig. 1, ‘Binomial_bayNorm’), or sampled from estimates obtained with a modified version of the Splatter package (Fig. 1, ‘Binomial_Splatter’, see Supplementary Note S2; Zappia *et al.*, 2017). These were compared with simulations generated with the original Splatter package which is based on the Gamma-Poisson distribution (Zappia *et al.*, 2017). Note that in Splatter, scaling factors are multiplicative to the Gamma distribution’s mean. In bayNorm, however, the cell-specific capture efficiencies, which act as scaling factors, are set as the probability parameter of the binomial model. Mean-variance relationship and mean-dropout relationship are two important features in scRNA-seq data. Several models have been proposed to explain these phenomenons (Anders and Huber, 2012; Hicks *et al.*, 2018; Kharchenko *et al.*, 2014; Pierson and Yau, 2015; Andrews and Hemberg, 2018a). The binomial model used in simulation (‘Binomial_bayNorm’) can better capture both relationships than Splatter (Fig. 1a–c).

Moreover, a parameter free approximation based on the binomial model predicted the dropout fraction to depend on an exponential of the negative mean expression (see Supplementary Note S1). This function produced a very close fit to the experimental data providing additional support for our choice of the binomial model (Fig. 1c). Notably, the Binomial_bayNorm simulation protocol using inferred gene-specific priors together with cell-specific parameters (*β_j_*) was the only one that recovered the distribution of dropout rates per gene observed in experimental data (Fig. 1d). Finally, the results presented on Figure 1b–e could be replicated consistently using several additional experimental scRNA-seq datasets (Supplementary Figs S2–S7).

The datasets discussed so far were obtained based on UMIs experimental protocol (Islam *et al.*, 2011). Datasets obtained without using UMIs are less likely to be well described by the binomial distribution. Accordingly, their dependence of dropout fractions on the mean expression has been reported to be more complex than in UMI-based datasets (Andrews and Hemberg, 2018a). We investigated this issue further and found that a simple scaling of non-UMI raw data by a constant factor produced a reasonable match to the binomial model (Supplementary Fig. S9; see Section 2). This scaling factor can be interpreted as the average number of times original mRNA molecules were sequenced after PCR amplification. This indicates that, provided appropriate scaling, non-UMI datasets are also compatible with the bayNorm model. Importantly, as bayNorm recovers dropouts rates successfully in both UMI-based and non-UMI protocols without the need of specific assumptions, we conclude that invoking zero-inflation models is not required to describe scRNA-seq data. Consistent with this, the differences in mean expression levels of lowly expressed genes observed between bulk and scRNA-seq data, which were suggested to be indicative of zero-inflation, were recovered by our simulated data using the binomial model only (Supplementary Fig. S10; Hicks *et al.*, 2018).

We note that the ability of simulation protocols to recover the statistics of experimental data depended intimately on the value of cell-specific capture efficiencies (*β_j_*). We used different ways to estimate *β* (spike-in, Scran scaling factors, trimmed means, or housekeeping genes; see Supplementary Fig. S8) together with different $\bar{\beta }$ in the Binomial_Splatter simulation protocol. We found that changes in *β_j_* values affected recovery of the distribution of dropout rates per cell. (Supplementary Fig. S8). In particular, we found that the use of spike-in controls or of housekeeping reference gene expression levels did not improve estimates of capture efficiencies (Supplementary Fig. S8c–f). Altogether, this analysis demonstrates that accurate statistics of experimental scRNA-seq data can be consistently retrieved using the binomial model and empirical Bayes estimation of gene expression parameters implemented in bayNorm along with accurate estimates of cell-specific capture efficiencies.

### 3.2 Recovery of true gene expression distributions and gene–gene correlation from scRNA-seq data

**Fig. 2.**
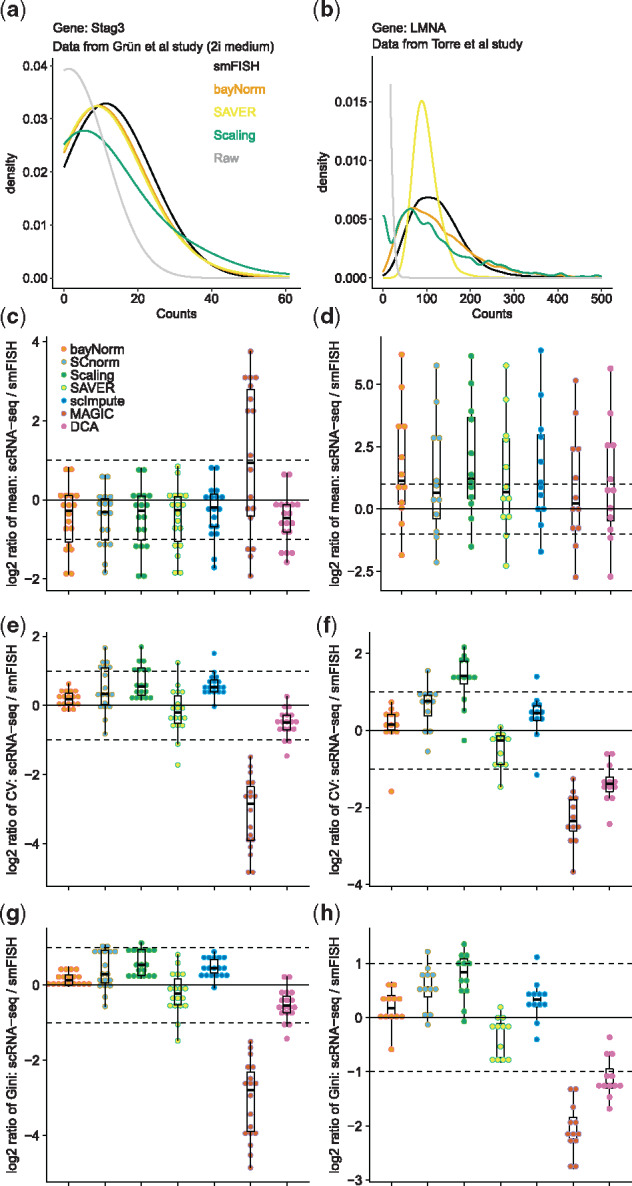
bayNorm recovers distributions of gene expression observed by smFISH. (**a**) Stag3 mRNA distribution for cells grown in 2i measured by smFISH or by scRNA-seq and normalized with different methods (from Grün study). ‘Raw’ denotes unnormalized scRNA-seq data. (**b**) As in (a) for the *LMNA* gene (from Torre study). Legend as in (a). Smoothing bandwidth is 10 for every method shown in (a and b). (**c**) Log_2_ ratio between the means of scRNA-seq measurements for 18 genes normalized by different methods and their matched smFISH measurements (from Grün study). (**d**) As in (c) using 12 genes (Torre study). (**e**) Log_2_ ratio between the CV of scRNA-seq measurements for 18 genes normalized by different methods and their matched smFISH measurements (from Grün study). (**f**) As in (e) using 12 genes (from Torre study). (**g**) Log_2_ ratio between the Gini coefficients of scRNA-seq measurements for 18 genes normalized by different methods and their matched smFISH measurements (from Grün study). (**h**) As in (c) using 12 genes (from Torre study). For the bayNorm and SAVER normalized datasets, 20 or 5 samples were generated from posterior distributions for the Grün and the Torre studies, respectively. All normalized datasets except bayNorm and the Scaling method have been divided by the $\bar{\beta }$ value used in bayNorm procedure. For this analysis smFISH data were normalized for variation in total transcript numbers using either cell size measurements (Grün study) or expression levels of a house keeping gene (Torre study) as detailed in see Supplementary Note S4

Single-cell RNA-seq provides a unique opportunity to study stochastic cell-to-cell variability in gene expression at a near genome-wide scale. However, doing this requires normalization approaches able to retrieve from scRNA-seq data transcripts levels matching quantitatively *in vivo* mRNA numbers (Torre *et al.*, 2018). bayNorm imputes drop-outs that are a result of low capture efficiency using its Bayesian approach (see Supplementary Fig. S25). However as bayNorm posterior models the original counts in the cell bayNorm should be effective in inference of the full transcript distributions. With this in mind, we evaluated bayNorm performance in reconstructing true gene expression levels from a series of experimental scRNA-seq datasets that contained matched single molecule fluorescence *in situ* hybridization (smFISH) measurements for a series of genes. We used mean capture efficiencies $\bar{\beta }$ estimated directly from smFISH together with gene-specific priors informed by the sequencing data (Supplementary Fig. S11). After bayNorm normalization, scRNA-seq counts reproduced accurately count distributions obtained by smFISH for several mRNAs (Fig. 2a and b). All methods captured mean smFISH counts across different genes well (Fig. 2c and d). However, noise in gene expression (coefficient of variation, CV) and expression dispersion (Gini coefficient) measured by smFISH were better captured by bayNorm compared with normalization by scaling or by several recent normalization and imputation methods (Fig. 2e–h; Bacher *et al.*, 2017; Eraslan *et al.*, 2019; Huang *et al.*, 2018; Li and Li, 2018; van Dijk *et al.*, 2018). bayNorm’s good performance could also be confirmed in a series of simulation studies (Supplementary Fig. S12).

Estimation of gene–gene correlations is essential in network inference from scRNA-seq data. As the bayNorm prior, assumes no correlation between genes, bayNorm could underestimate the correlations. We used Torre study that contains smFISH data on gene–gene correlations to illustrate that bayNorm gene-specific priors indeed underestimate the gene-gene correlation (Supplementary Fig. S26). In comparison the adjusted SAVER correlation estimates tend to overestimate the gene–gene correlation for most pairs of genes (Supplementary Fig. S26). We believe this is due to pooling information across genes in the same cell in SAVER’s empirical Bayes approach. However, bayNorm does not inflate gene–gene correlations as observed for some imputation methods (Andrews and Hemberg, 2018b). In summary, bayNorm combined with gene-specific priors inferred directly from the scRNA-seq data, retrieves gene expression variability and gene–gene correlations matching smFISH data.

### 3.3 bayNorm enables accurate and sensitive DE analysis

Differential gene expression analysis in scRNA-seq studies is challenging as several factors including variability in capture efficiencies, dropout rates, sequencing depth and experimental batch effects can introduce significant, yet spurious, DE signal. Normalization and imputation approaches have, therefore, a significant impact on the sensitivity and accuracy of DE analysis protocols. Two features of the bayNorm approach have the potential to improve the performance of DE analysis. First, bayNorm posterior distribution of original counts maintains the uncertainty resulting from small capture efficiencies and could therefore reduce false positive DE discovery rates (Pimentel *et al.*, 2017). Second, the use of priors specific to each group of cells compared in the DE analysis could increase true positive discovery rates. With this in mind, we have assessed bayNorm performance in DE analysis using several experimental scRNA-seq datasets and compared it with other normalization and imputation methods. To identify DE genes we use model-based analysis of single-cell transcriptomics (MAST) (Finak *et al.*, 2015), which performs well in terms of false positives rates, precision and recall (Jaakkola *et al.*, 2016). MAST was first applied to individual sample from the bayNorm posterior distribution (3D array, Supplementary Fig. S1). Differentially expressed genes were then called based on the median of Benjamini-Hochberg adjusted *P*-values of the individual samples (Benjamini and Hochberg, 1995).

**Fig. 3.**
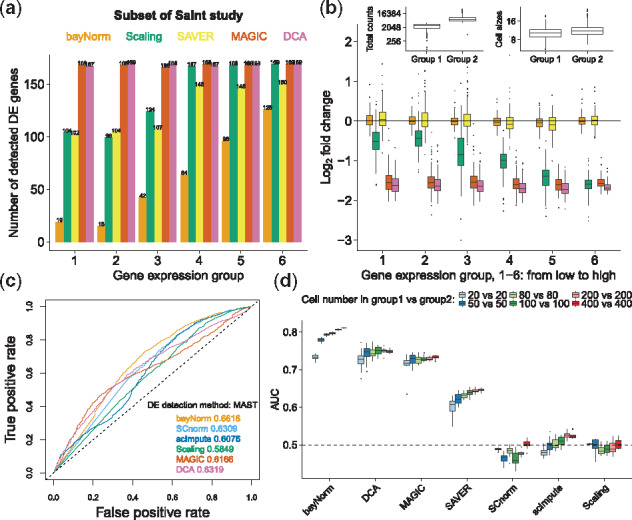
bayNorm enables robust and sensitive DE analysis. (**a**) Number of differentially expressed genes between the 1000 cells with the highest and the 1000 cells with the lowest total counts in Saint study (Saint *et al.*, 2019). DE genes were called using the MAST package ($P_{\text{MAST}}<0.05$) and plotted for six groups of genes with increasing mean expression (1—low to 6—high). (**b**) log_2_ fold-change from (a). Inset shows box plots of total count and cell sizes (as measured in Saint *et al.*, 2019) in the two groups, illustrating lack of strong correlation between scRNA-seq raw total count and cell size. (**c**) DE analysis using MAST for different normalization methods (Islam study) using a benchmark list of DE genes obtained from matched bulk RNA-seq data Ye *et al.* (2019). (**d**) DE analysis using data from Soumillon study (Soumillon *et al.*, 2014). 20, 50, 80, 100, 200 or 400 cells were selected randomly from two groups of stage-3 differentiated cells at day 0 (D3T0) or day 7 (D3T7). A list of DE genes obtained from matched bulk RNA-seq data was used as a benchmark (1000 genes with the largest magnitude of log fold-change between the D3T0 and D3T7 samples, Ye *et al.*, 2019). For bayNorm and SAVER, 3D arrays were used

As mentioned earlier, differences in capture efficiencies between cells is a source of technical variability that could affect DE analysis. To test bayNorm’s ability to correct for this bias, we selected the 1000 cells with the highest and lowest capture efficiencies based on total counts in a recent UMI-based scRNA-seq study of fission yeast with cell size measurements (Saint *et al.*, 2019). We then applied bayNorm to the 2000 cells using global prior estimation by pooling information across the two groups (see Section 2). In this design, the two groups of cells differ based on their capture efficiencies while the cell size in two groups is not markedly different (Fig. 3b, inset). Therefore, significant DE is not expected. Figure 3a shows the number of genes called differentially expressed as a function of increasing average expression levels using a series of normalization and imputation methods. bayNorm normalized data show almost no differentially expressed genes, outperforming all the other methods. Moreover, log_2_ gene expression ratios between cells of the two groups were consistently close to zero, confirming bayNorm ability to correct for biases inherent to different capture efficiencies in UMI-based datasets (Fig. 3b).

Sequencing depth is another parameter affecting DE analysis especially because it impacts on the dropout rates of lowly expressed genes. Moreover, differences in sequencing depth are likely to affect levels of capture efficiencies, especially for non-UMI datasets where PCR biases are not accounted for. To assess bayNorm’s ability to correct for this source of bias, we used a benchmark dataset published by Bacher *et al.* (2017) that consists of non-UMI-based scRNA-seq data for two groups of cells isolated from a single culture and sequenced to a depth of either 1 or 4 million reads per cell. bayNorm and other imputation methods performed well in this setting (Supplementary Fig. S13). Finally, bayNorm corrected robustly for variability in sequencing depth when applied to a series of simulated datasets (Supplementary Figs S14 and S15; Bacher *et al.*, 2017).

We have shown that bayNorm is efficient at removing spurious DE from scRNA-seq data caused by variability in capture efficiencies and sequencing depth. We next explored bayNorm performance in supporting sensitive and robust detection of genes truly regulated between samples. To do this, we used two experimental scRNA-seq datasets (Islam *et al.*, 2011; Soumillon *et al.*, 2014) and lists of benchmark DE genes derived from matched bulk RNA-seq data (Jaakkola *et al.*, 2016; Ye *et al.*, 2019). To maximize sensitivity, we used priors specific to each groups of cells in the comparison (we call this design ‘local priors’). With the first dataset, bayNorm normalized data generated an area under the curve (AUC) value as high as other normalization methods demonstrating that the approach supports sensitive DE detection (Fig. 3c). Analysis of the second dataset (UMI-based) (Soumillon *et al.*, 2014) further confirmed this observation with bayNorm performing better than all other methods (Fig. 3d). Importantly, bayNorm’s performance did not depend on the number of cells in each group, except for groups with very low numbers of cells (Fig. 3d andSupplementary Fig. S16). Finally, using a series of simulated datasets, we explored situations where the compared groups have different mean capture efficiencies and found that bayNorm supported robust DE detection in all cases (Supplementary Fig. S17).

Three important parameters should be considered before bayNorm normalization: (i) the choice of priors, (ii) the choice of average capture efficiencies $\bar{\beta }$, iii) the choice of bayNorm output format (2D versus 3D array). Prior parameters can be either estimated for all cells across groups (global) or within each group (local). Since priors are gene specific, applying bayNorm across homogeneous cells (i.e. using global prior) allows for mitigating technical variations (Supplementary Fig. S18a and b). On the other hand, using priors estimated ‘locally’ within each group amplifies differences in signals between heterogeneous groups of cells increasing sensitivity (Supplementary Fig. S18c and d). Average capture efficiencies $\bar{\beta }$ are specific to each scRNA-seq protocol and reflect their overall sensitivity. This value represents the ratio of the average number of mRNA molecules sequenced per cell to the total number of mRNA molecules present in an average cell. It is not always easy to determine as quantitative calibration methods such as smFISH are not widely used, and approaches based on spike-in controls have shortcomings (Vallejos *et al.*, 2017). We investigated the impact of inaccurate estimation of *β* on biases in DE detection. Critically we found that DE results based on bayNorm normalized data are not affected significantly by a 2-fold change of $\bar{\beta }$ (Supplementary Figs S20 and S21). Finally, bayNorm output consists of either samples from its posterior distributions (3D array) or the modes/means of these distributions as point estimates (2D arrays). For DE analysis using MAST, 3D array outputs reduces false positive rates (FPRs) but 2D array outputs perform slightly better in terms of AUC (Supplementary Fig. S18c and d). Supplementary Figure S19 shows DE results for two other non-parametric methods: reproducibility optimized test statistic (Elo *et al.*, 2008) and Wilcoxon test (Jaakkola *et al.*, 2016). Both approaches perform equally well with 3D arrays but show variable results when applied to 2D arrays with the Wilcoxon test performing less well. In summary, our analysis demonstrates that in addition to correcting for technical biases, bayNorm also supports robust and accurate DE analysis of a wide range of experimental and simulated scRNA-seq datasets.

### 3.4 bayNorm correction of experimental batch effects

scRNA-seq protocols are subject to significant experimental batch effects (Tung *et al.*, 2017). bayNorm can mitigate batch effects in two ways. First, as described above, bayNorm efficiently corrects for differences in capture efficiencies which is a pervasive source of batch-to-batch variability (Hicks *et al.*, 2018). Second, the use of bayNorm data-informed priors is an efficient way to mitigate batch variation by estimating prior parameters across different batches but within the same biological condition.

**Fig. 4.**
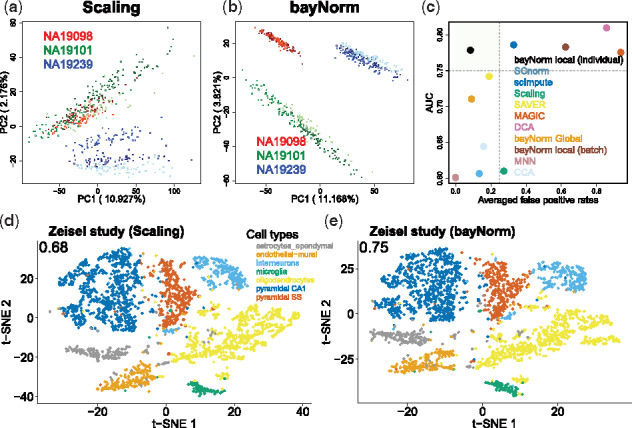
Batch effect correction and cell type identification (**a** and **b**) each color represent a different cell line derived from a different individual. Color shades represent different batches within a line/individual. (**c**) Differentially expressed genes were called between lines NA19101 and NA19239 as well as different batches within each line (seven pair of comparisons in total). FDRs were averaged across the seven pairs. The vertical and horizontal dashed lines represent 0.25 and 0.75 indicative cutoffs, respectively. bayNorm was applied either across batches but within lines [‘bayNorm local (individual)’] or across all cells (‘bayNorm global’) or within each batch [‘bayNorm local (batch)’]. Global gene-specific prior parameter estimation across all cells results in clear clusters of different cell types compared with Scaling normalization using the data from Zeisel *et al.* (2015). t-SNE plots are shown based on Scaling normalization (**d**) and bayNorm (**e**). The clustering performance is quantified by Jaccard index (the value reported at the top left of each panel). For bayNorm, 1 sample of 3D array was used. (Color version of this figure is available at *Bioinformatics* online.)

To investigate bayNorm’s performance for batch effect correction we use data from the Tung study, where there are three batches for each of three individuals (Tung *et al.*, 2017). We first used priors calculated within each individual, but across batches [bayNorm local (individual)]. This strategy allows for maintaining differences between individuals while minimizing batch effects as illustrated in Figure 4a and b (also see Supplementary Fig. S22). To quantify the result, we defined the ratio of the number of DE genes (detected between each pair of batches within the same individual, adjusted $P_{\text{MAST}}$ < 0.05) and the total number of genes (13 058) to be the FPRs (Supplementary Fig. S23). In parallel, we tested whether bayNorm also maintained differences between individuals. To do this, we detected DE genes between the iPSC lines NA19101 and NA19239 and compared it with a benchmark list of 498 DE genes (Ye *et al.*, 2019). Efficient batch effect correction is expected to minimize FPR while maximizing AUC values of DE detection between individuals. Using bayNorm with ‘within individual’ local priors (estimated across different batches within the same line) outperformed other methods in terms of correcting batch effects while maintaining meaningful biological information. As expected, using global priors (estimated across batches and individuals, bayNorm global) preserves low FPR, but reduces AUC significantly. Finally, using ‘within batch’ local priors [bayNorm local (batch)] result in higher FPRs, which is also expected.

Another common use of scRNA-seq data in heterogeneous systems is to identify different cell types by clustering cells in an unsupervised manner. The Zeisel study provides a dataset where each cell is assigned to a specific cell type based on lineage markers expression, which can therefore be used as gold standard Zeisel *et al.* (2015). In Figure 4d and e, we compared 2D t-distributed stochastic neighbor embedding (t-SNE) plots (van der Maaten and Hinton, 2008) of cells based on the scaling and bayNorm methods, respectively. We used the Seurat package for cell clustering (Butler *et al.*, 2018). The Jaccard index was calculated for each method using ‘cluster_similarity’ function from R package ‘clusteval’. This analysis illustrates how bayNorm with global priors can preserve clustering of different cell types as well as the scaling methods. Moreover, this observation was confirmed using two additional datasets, where cell type annotation was not provided (Baron *et al.*, 2016; Chen *et al.*, 2017). There, we used cell labels determined based on scaling normalized data as references (see Supplementary Fig. S24).

## 4 Discussion

We introduced bayNorm, a versatile Bayesian approach for implementing global scaling that simultaneously provides imputation of missing values and true counts recovery of scRNA-seq data. Bayesian methods have been applied to different aspects of RNA-seq data analysis before (Hardcastle and Kelly, 2010; Huang *et al.*, 2018; Kharchenko *et al.*, 2014; Vallejos *et al.*, 2015). We showed that using the binomial model and an empirical Bayes approach to estimating gene expression priors across cells results in simulated data almost identical to experimental scRNA-seq measurements. Importantly, this suggests that zero-inflated models are not required to explain the frequency of dropout observed in scRNA-seq (see also Svensson, 2019; Soneson and Robinson, 2018). Although designed initially for UMI-containing scRNA-seq protocols, a simple scaling factor makes bayNorm applicable to non-UMI data as well. This flexibility will allow using this approach with most present scRNA-seq datasets. We showed using datasets that combine smFISH and scRNA-seq, that bayNorm is accurately recovering true gene expression across a wide range of expression levels. This approach could therefore be particularly useful for quantitative analysis of more difficult scRNA-seq datasets, such as those generated from small quiescent cells or microbes, for instance. In fact, we have recently used bayNorm successfully in the first scRNA-seq study of fission yeast (Saint *et al.*, 2019).

One of the most powerful features of bayNorm is its use of gene expression priors directly calculated from gene expression values across cells. We showed that grouping cells according to experiment design or phenotypic features increased significantly the robustness and sensitivity of DE analysis. This removes almost completely the sequencing depth and capture efficiency biases, and reduces batch effects. Critically, this approach preserved accurate and sensitive detection of benchmark DE genes in contrast to some recently developed methods of batch correction. Also, where there is no prior knowledge available for cell types, using a global approach does not affect clustering of cell types. The clustering results could be improved if priors are iteratively improved using a method of simultaneous normalization and clustering as proposed in Prabhakaran *et al.* (2016).

Bayesian methods have been applied to different aspects of RNA-seq data analysis before (Hardcastle and Kelly, 2010; Huang *et al.*, 2018; Kharchenko *et al.*, 2014; Prabhakaran *et al.*, 2016; Vallejos *et al.*, 2015). The approach most related to bayNorm is taken by SAVER, which uses a Poisson-Gamma model and pooling information across genes for true count recovery (Huang *et al.*, 2018). In contrast, bayNorm uses a binomial model of mRNA capture as likelihood and achieves similar or improved performance relative to SAVER on real and simulated data (Figs 2–4).

Accurate estimation of cell capture efficiencies (or scaling factors) is central to most scRNA-seq normalization methods including bayNorm. Interestingly, we observed that the choice of cell-specific capture efficiencies affect how closely simulated data recovers statistics of real data. We therefore propose that comparison of drop-out rates per cell in simulated datasets and experimental data could be used as a tool to inform appropriate choice of global scaling factors and mean capture efficiency estimates. The option to tailor bayNorm priors based on phenotypic information about cell subpopulations will be a powerful asset for discovery of gene expression programs associated with specific phenotypic features of single cells such as cell size (Saint *et al.*, 2019). Finally, the concepts and mathematical framework behind bayNorm will be useful if combined with other emerging theoretical approaches such as deep learning (for instance Ding *et al.*, 2018; Eraslan *et al.*, 2019; Grønbech *et al.*, 2018; Lopez *et al.*, 2018; Wang and Gu, 2018). Overall, bayNorm provides a simple and integrated solution to remove the technical biases typical of scRNA-seq approaches, while enabling robust and accurate detection of cell-specific changes in gene expression. bayNorm has been made freely available as an R package (https://bioconductor.org/packages/release/bioc/html/bayNorm.html) released in Bioconductor (Gentleman *et al.*, 2004).

## Supplementary Material

btz726_Supplementary_Data
