# Supplementary material for: bayNorm: Bayesian gene expression recovery, imputation and normalization for single-cell RNA-sequencing data
Source: Bioinformatics. 2019 Oct 4;36(4):1174–81. doi: 10.1093/bioinformatics/btz726 (PMC7703772; doi:10.1093/bioinformatics/btz726)
Supplement: btz726_Supplementary_Data [file btz726_supplementary_data.pdf]

bayNorm: Bayesian gene expression recovery, imputation and normalisation for single cell RNA-sequencing data

Wenhao Tang, François Bertaux Philipp Thomas Claire Stefanelli, Malika Saint, Samuel Marguerat and Vahid Shahrezaei

Supplementary Information

Supplementary Note 1: Binomial model and estimation of parameters in bayNorm

Estimation of capture efficiencies

Cell specific capture efficiency β_j and global scaling factor (s_j) are closely related. The global scaling factors for the single cell RNA-seq data corrects technical variations including differences in capture efficiency and also in the non-UMI protocols for differences in amplification and sequencing depth [1]. Also, global scaling factors correct for biological variations due to difference in transcript content and cell size. In the absence of cell size (RNA content) information, we assume that the cell specific capture efficiencies are proportional to global scaling factors that can be estimated using different methods (see below). Therefore, we have:

$$\beta_j = (s_j/\bar{s})\bar{\beta} \tag{1}$$

$\bar{\beta}$, a scalar, is an estimate of global mean capture efficiency across all cells, which ranges between 0 and 1. We note that using global capture efficiencies in normalisation removes both technical and biological variations in single cells and produces estimates of transcript counts corrected for changes in cell size and total transcript count. The bayNorm normalised output can be thought of estimate of original transcript count in the cell if the cell had an average size (or total transcript content).

There are two different methods for estimating $\bar{\beta}$ and β_j:

1. If spike-ins or smFISH data are available they can be used to estimate capture efficiencies. We can either divide the total number of observed spik-ins in each cell by the total number of input spike-ins, or we can fit a linear regression ([2]) to estimate the cell specific β_j. If smFISH data is available, we can fit a linear regression between the mean expression of raw data (response variable) and the mean expression of the smFISH data (explanatory variable). The coefficient of the explanatory variable can be used as $\bar{\beta}$ ([3]).

2. The raw data itself can be directly used for estimation of cell specific global scaling factors (s_j). Then equation 1 and an estimate of $\bar{\beta}$ can be used to estimate β_j. There are different methods available for estimation of global scaling factors. Some were developed for bulk RNA-seq data ([4, 5]) and some are specific to scRNA-seq data[6, 7]. The value of $\bar{\beta}$ depends on the protocol used and can be batch dependent. For example, for Droplet based protocol, it is about 0.06 ([2]) or 0.12 ([8]). $\bar{\beta}$ can also be estimated by spike-ins or smFISH data as explained above.

We finally note that estimates of capture efficiency discussed above will assume cells have similar original transcript content. Therefore, the bayNorm outputs estimates of original transcript counts

for a typical cell, which is corrected for variation in cell size and transcript content. This is usually desirable for down-stream analysis such as DE detection. However, if one is interested in absolute original count and has additional information such as cell size or total transcript content per cell, the capture efficiencies can be appropriately rescaled for this purpose.

Binomial distribution and dropout probability

The binomial model of capture in scRNA-seq predicts the dropout rate for a particular gene:

$$\Pr(x_{ij} = 0 | x^0_{ij}, \beta_j) = (1 - \beta_j)^{x^0_{ij}},$$

in a given cell j. Across a group of non-homogeneous cells, we may approximate this expression by

$$(1 - \bar{\beta})^{(\bar{x}/\bar{\beta})}$$

For small $\bar{\beta}$ this expression tends to $\Pr(x = 0) = \exp(-\bar{x})$. In dropout vs mean expression (dropout-mean) (Figure 1c, Sup Figures S2c, S3c, S4c, S5c, S6c and S7c), the line "$\exp(-\bar{x})$" follows the lower limit of the trend. We note that a Poisson model of RNA-seq that is used by several authors also predicts dropout rates to be $\Pr(x = 0) = \lambda^0/0! \exp(-\lambda) = \exp(-\lambda)$, where $\lambda = \bar{x}$ ([9, 10, 11]).

To further show that Binomial distribution can capture the relationship between dropout rates and mean expression, we simulated data based on real experimental data ([2, 12, 13]) by adapting simulation protocols proposed in the R package Splatter ([10]). The details about the simulation procedure can be found in the supplementary. The resulting dropout-mean plot of simulated data based on Binomial model is very close to that of the real scRNA-seq data for UMI-based protocols. As shown in the Supplementary Figures S2c, S3c, S4c, S5c and S6c, the dropout-mean trend of UMI data is close to the asymptotic line "$\exp(-\bar{x})$" ("Binomial_Splatter" and "Binomial_bayNorm" simulated data perform similar to each other and the real experimental data). data based on real experimental data ([2, 12, 13]) as discussed in the results and supplementary. The resulting dropout-mean plot of simulated data based on Binomial model is very close to that of the real scRNA-seq data for UMI-based protocols.

Estimation of prior parameters

Maximisation of marginal likelihood

Using an emperical bayes approach, one can use the maximisation of marginal likelihood distribution of the observed counts across cells to estimate prior parameters ([14]). Let M_i denotes the marginal likelihood function for the ith gene across cells. Assuming independence between cells, the log-marginal likelihood for the ith gene is

$$\log M_i = \sum_{j=1}^{Q} \log \Pr(x_{ij} | \mu_i, \phi_i, \beta_j), \tag{2}$$

where $\Pr(x_{ij} | \mu_i, \phi_i, \beta_j)$ is the Negative Binomial (see Methods). Maximizing of Eq. (2) yields the pair (μ_i, ϕ_i).

The above optimization needs to be done for each of the P genes. We refer to the ϕ and/or μ estimated by maximizing marginal likelihood as BB estimates for convenience, because bayNorm utilizes spectral projected gradient method (spg) from the R package named "BB". Optimizing the marginal likelihood with respect to both μ and ϕ (2D optimization) is computationally intensive. If we had a good estimate μ, then we could optimize the marginal likelihood with respect to ϕ alone, which would be much more efficient.

Method of Moments

A heuristic way of estimating μ_i and ϕ_i is through a variant of the Method of Moments. The first step is to do a simple normalization of the raw data, to scale expressions given the cell specific capture efficiencies (β_j). The simple normalized count x_{ij}^s is calculated as following:

$$x_{ij}^s = x_{ij}/\beta_j, \tag{3}$$

where the numerator of the scaling factor of x_{ij} is obtained by taking the average of scaled total counts across cells.

Based on simple normalized data, we are able to estimate prior parameters μ and ϕ of the Negative Binomial distribution using the Method of Moments Estimation (MME), which simply equates the theoretical and empirical moments. This estimation method is fast and simulations suggests it provides good estimates of μ but the drawback is that the estimation of ϕ show a systematic bias (see Supplementary Figure S27 a-b).

The combined method

Based on simulation studies (Supplementary Figure S27), the most robust and efficient estimation of μ and ϕ can be obtained using the following combined approach, which is the default setting in bayNorm:

1. We apply the MME method for each gene to obtain MME estimated μ and ϕ.
2. The BB estimated ϕ does not suffer from the bias observed in the MME estimated ϕ, but estimates are less robust (many estimates are at the upper boundary of the search space; Supplementary Figures S27 c-d). So, we find adjusting the MME estimated ϕ by a factor which can be estimated by fitting a linear regression between MME estimated ϕ and BB estimated ϕ works best (Supplementary Figures S27 c-d). This adjusted MME estimated ϕ together with the MME estimated μ are used in bayNorm by default.

The combined method results in a better estimation of dispersion parameter in Negative Binomial distribution than SAVER, DESeq2 and BASiCS[9, 4, 7, 27] (Supplementary Figures S28).

Cells are grouped together for prior estimation, based on cell-specific attributes (C_j). Prior estimation can be done over all cells irrespective of the experimental condition. We refer to this procedure as "global". Alternatively, suppose that there are multiple groups of cells in the datasets and we have reasons to believe each group could behave differently. Then we can estimate the prior parameters "μ and ϕ" within each group respectively (within groups with the same C_j value). We refer to this procedure as "local". Estimating prior parameters across a certain group of cells based on "global" procedure allow for removing potential batch effects. Multiple groups normalization based on "local" procedure allows for amplifying the inter-groups' differences while mitigating the intra-group's variability, which is suitable for DE detection.

Supplementary Note 2: two simulation protocols with Binomial distribution

"Binomial_Splatter" simulation protocol

We adapted the simulation protocol proposed in the R package Splatter[10] but made two main modifications to that protocol:

1. We do not multiply the mean of the Gamma distribution by the library size factors. Instead, we add cell specific factors (capture efficiencies β_j) at the last stage of simulation: Binomial step.

2. Unlike Splatter, we do not model the dropout rates explicitly. Instead we dropouts are the result of Binomial downsampling at the last stage of the simulation, which leads to a dropout vs mean expression relationship in the simulated data very similar to the one of experimental data (Supplementary Figures S2c, S3c, S4c, S5c and S6c).

The details of the simulation procedure are as follow:

1. We simulate a vector of base mean expressions λ'_i such that

$$\lambda'_i \sim \text{Gamma}(\text{shape} = \alpha_1, \text{rate} = \alpha_2)$$

.

2. We simulate a vector of outlier factors ψ_i such that $\psi \sim \ln\mathcal{N}(\mu^0, \sigma^0)$. For a proportion π^0 of genes, we multiply the base mean expression by outlier factors: $\lambda^0_i = 1^0_i \lambda_i \psi_i \text{median}(\lambda'_i) + (1 - 1^0_i)\lambda'_i$, where $1^0_i \sim \text{Ber}(\pi^0)$. The above two steps are the same as those implemented in Splatter.

3. In the simulations with differential expression (SIM DE), the mean expression λ^0_i for the two groups are the same except that in the first group, we multiply λ^0_i by a vector of DE factors simulated from the log normal distribution. Conversely, in SIM noDE study, no DE genes were simulated.

4. Then, $\lambda_i \sim \text{Gamma}(1/B_i^2, \lambda^0_i B_i^2)$, where $B_i = (d + 1/\sqrt{\lambda^0_i})(df/\chi^2(df))^{1/2}$ stands for the Biological Coefficient of Variation (BCV), and d is common dispersion. $1/B^2$ corresponds to the dispersion parameter ϕ in the Negative Binomial distribution.

5. Then the true count $x^0_{ij} \sim \text{Poi}(\lambda_i)$. So far, no cell-specific factors have been taken into consideration.

6. Then a vector of cell specific capture efficiencies β_j needs to be specified in the simulation. When we compare the simulated data with the real data, the capture efficiencies estimated from the real data are used in the simulation. In all simulation studies, β is simulated from the log normal distribution and normalized to a specific mean capture efficiency (either 0.05 or 0.1).

7. Lastly, we implement the binomial step to obtain the observed count (binomial downsampling):

$$x_{ij} \sim \text{Binom}(x^0_{ij}, \beta_j)$$

"Binomial_bayNorm" simulation protocol

Unlike previous simulation protocol where gene expression parameters were simulated from a specific distribution with several estimated parameters, here we use gene specific priors estimated by bayNorm together with β_j to conduct gene and cell specific simulation. So, this method produces exactly simulated data of the same size as the real data ($P \times Q$).

Let μ_i and ϕ_i be the estimated mean expression and dispersion parameter obtained by bayNorm for the ith gene. Firstly a mean expression matrix (λ'_{ij}) which is of the same dimension as the real data is created, such that $\lambda'_{ij} = \mu_i$ across j. Then sampling from the following distributions leads to the simulated data:

$$\begin{aligned}
&\lambda_{ij} \sim \text{Gamma}(\text{shape} = \phi_i, \text{scale} = \lambda'_{ij}/\phi_i) \\
&x^0_{ij}(\text{true count}) \sim \text{Poi}(\lambda_{ij}) \\
&x_{ij}(\text{observed count}) \sim \text{Binom}(x^0_{ij}, \beta_j)
\end{aligned} \tag{4}$$

Parameter estimation from the real data ("Binomial_Splatter" simulation protocol)

The parameter estimation methods used in the simulation are basically the same as those in Splatter, except that the input raw data are scaled by β_j, before fitting the mean expression of the scaled data using Gamma distribution to estimate α_1 and α_2.

The estimation of other parameters like π^0, μ^0, σ^0, B_i were achieved based on library size normalized data as also implemented in Splatter. Non-UMI based data were scaled and rounded before parameters were estimated as explained before.

Supplementary Note 3: Simulation studies using the "Binomial_Splatter" simulation protocol

We estimated parameters from the Klein and Bacher studies (92 H1-4M hESCs) and then generated 6 simulated datasets for comparing different normalization methods for their performance in correcting different capture efficiencies (study without DE genes), and in DE genes detection.

Two simulated datasets are generated without DE genes (homogeneous cells across two groups, but different mean capture efficiencies):

SIM noDE study I (Using parameters estimated from the Klein study): The mean capture efficiencies of the two groups are 0.1 and 0.05 respectively. Simulated data was used in: Supplementary Figures S14, S18a-b, S19b.

SIM noDE study II (Using parameters estimated from 92 H1-4M hESCs of the Bacher study): The mean capture efficiencies of the two groups are 0.1 and 0.05 respectively. Simulated data was used in: Supplementary Figures S15, S19c.

Simulation SIM DE studies are all based on parameters estimated from the Klein study.

SIM DE study I: The mean capture efficiencies for the two groups are both 0.1. Simulated data was used in: Supplementary Figures S12a,e,i, S17a, S18c,d, S21a and S27.

SIM DE study II: The mean capture efficiencies for the two groups are 0.05 and 0.1 respectively. Simulated data was used in: Supplementary Figures S12b,f,j, S17b, S18c,d, S21b and S28.

SIM DE study III: The mean capture efficiencies for the two groups are 0.1 and 0.05 respectively. Simulated data was used in: Supplementary Figures S12c,g,k, S17c, S18c,d, and S21c.

SIM DE study IV: The mean capture efficiencies for the two groups are both 0.05. Simulated data was used in: Supplementary Figures S12d,h,l, S17d, S18c,d, and S21d.

Genes with 0 counts across two groups were filtered out at the very beginning. No cells were filtered out. Belows are details about parameter settings used in the simulation studies which were estimated from the raw data as discussed above.

Parameters estimated from the Klein study: Most parameters were estimated from the Klein study except β. For each group, 10000 genes and 100 cells were simulated. The base mean expression for both groups were simulated from the Gamma distribution with $\alpha_1 = 1.889$ and $\alpha_2 = 0.1229$. $\pi^0 = 3\%$ across two groups. The outlier factors were simulated from the log normal distribution with μ^0 and σ^0 set to 2.3 and 0.75 respectively. BCV was calculated with $d = 0.12$ and $df = 105$.

The estimation of β for a specific real experimental data is discussed in the next Supplementary note.

In the SIM noDE study I and the SIM DE studies I-IV, two groups of 100 cells with 10000 genes were simulated using the above parameter settings. β_j are simulated from the log normal distribution within each group with mean and sd (log scale) set to 2.74 and 0.3908 respectively. Within each group, we normalized the β to either 0.1 or 0.05.

In the SIM DE I-IV studies, the DE factors in the first group are simulated from the log normal distribution with log scale mean and sd set to 1 and 0.5 respectively.

Parameters estimated from the Bacher study (based on 92 H1-4M hESCs, 4 million mapped reads per cell): Parameters were estimated from the raw data scaled by 20. $\alpha_1 = 0.4129$ and $\alpha_2 = 0.005766$. Outliers genes were simulated with $\pi^0 = 0.7\%, \mu^0 = 4.745$ and $\sigma^0 = 0.6027$. BCV was calculated with $d = 0.3113$ and $df = 7.6859$.

In the SIM noDE case study II, two groups of 100 cells with 10000 genes were simulated using the above parameter setting. β_j are simulated from the log normal distribution within each group with mean and sd (log scale) to be -2.276 and 0.6886 respectively. Within each group, we normalized the β to either 0.1 or 0.05.

Supplementary Note 4: Publicly available datasets and their preprocessing

Bacher study (non-UMI).

Single-cell RNA-seq expression data were downloaded from GEO GSE85917[15]. In this experiment, two groups of undifferentiated H1 hESCs were sequenced to a depth of 4 million mapped reads per cell and 1 million mapped reads per cell respectively. A similar experiment was done for H9 hESCs cells. The following filtering protocol was used in our study: spike-ins and genes which do not have at least 10 non-zero counts were removed. After filtering, each group of H1 cells had 92 cells and 13181 genes, while groups of H9 cells had 91 cells and 13195 genes. Since these are non-UMI based data, we divided the raw data by a factor 20 for the 4 million mapped reads group and 10 for the other group so that scaled and rounded raw data were closer to the theoretical dropout vs mean curve (Supplementary Figure S9 a-d).

In order to estimate β, we let the total counts of observed spike-ins in each cell be the scaling factors s_j, and then normalized to 0.1 (based on scaled ERCC data, see Methods). As discussed in the text, within a 2 fold window of mean β, the performance of bayNorm in terms of DE detection is consistent (Supplementary Figure S20 a). Since cells in the two groups are the same, prior parameters were estimated across two groups when applying bayNorm ("global priors"). 20 samples were generated to form 3D array in bayNorm. DE detection was performed in each sample and summarized as median of adjusted P-values.

Data was used in the Supplementary figures: S7, S9a-d, S13, S19a and S20a.

Islam study (non-UMI).

Raw data (48 ES cells and 44 MEF cells with 7284 genes) and a list of benchmark DE genes were kindly provided by Maria K. Jaakkola[16]. Genes which have zero expressions across all the 92 cells were removed in advance which left us with 5826 genes. Raw data was divided by a factor 10 before applying bayNorm on it as this is a non-UMI data and the scaled and rounded raw data is closer to the theoretical dropout vs mean curve.

For estimating β, scaling factors were estimated using scran[6] and were normalized to 0.03 (see Methods). The impact of different $\bar{\beta}$ can be found in Supplementary Figure S20 b. 20 samples were generated to form 3D array in bayNorm. DE detection was performed in each sample and summarized as median of adjusted P-values.

Data was used in the Figure 3c, Supplementary figures: S9e-f, S10e and S20b.

Patel study (non-UMI).

Raw data were stored in the R package "patel2014gliohuman"`https://github.com/willtownes/patel2014gliohuman`[17]. Single cell data were scaled by a factor 20 and rounded as this is a non-UMI data and the scaled and rounded raw data is closer to the theoretical dropout vs mean curve. Cells with total counts less than or higher than the tenth percentile of total counts across cells were filtered out. In addition, genes with mean expression less than 1 were also removed. β were estimated using scran[6] with parameter "positive=TRUE". The size factors were normalized to 0.06 (see Methods). Finally, cells with $\beta < 0.01$ were filtered out, leaving 590 cells and 5519 genes in the final datasets. We applied bayNorm on the scaled, rounded and preprocessed dataset. The estimated priors were used as input for Binomial_bayNorm simulation protocol (Supplementary Figure S10f).

Data was used in the Supplementary figures: S10f.

Tung study (UMI).

Filtered molecule count matrix as well as the code for estimating β using spike-ins were downloaded from the GitHub repository: `https://github.com/jdblischak/singleCellSeq` [13]. The list of benchmark DE genes was kindly provided by the author of R package DECENT[18]. Genes with 0 values across all three individuals were filtered out leaving 13058 genes in the final dataset. No cells were filtered out, resulting in 142, 201 and 221 cells for individuals NA19098, NA19101 and NA19239 respectively. 5 samples were generated to form 3D array in bayNorm. One of them was used in PCA plot.

Data was used in the Figure 4, Supplementary figures: S3-5, S8c-d, S10b-d and S22-23.

Grün study (UMI).

Single-cell RNA-seq expression data were downloaded from GEO GSE54695[3]. The smFISH data used in that paper was kindly provided by the author.

The downloaded data was transformed to transcript number. We adapted the code provided in the supplementary material of [19] to convert the data to UMI count. We followed the same criterion as [19] for filtering genes in the 2i and serum data respectively. After filtering, we kept 74 cells and 9377 genes for the 2i medium data. For the serum medium data, we kept 52 cells and 9440 genes. 20 samples were generated to form 3D array in bayNorm.

smFISH data were normalized by scaling factors which were calculated as cell sizes divided by mean of cell sizes.

Total number of input spike-ins was estimated by adapting the code provided in the supplementary information of [19]. We divided the total number of observed spike-ins in each cell by the total number of input spike-ins to obtain scaling factors. We used smFISH data to estimate $\bar{\beta}$ for single cells under 2i and serum medium respectively (0.1212 and 0.1187 respectively, Supplementary Figure S11a-b). To obtain β, we normalized scaling factors (see Methods) to the corresponding $\bar{\beta}$ within each one of the two datasets.

Data was used in the Figure 2a,c,e,g and Supplementary figures: S11a-b.

Klein study (UMI).

Single-cell RNA-seq expression data were downloaded from GEO GSE65525[2]. ES cells data at day 0 (933 cells with 24175 genes) were used for simulations. We did not filter out any genes or cells.

For estimating β, trimmed mean of each cell at 1% was used and was normalized to $\bar{\beta}$ set to 0.06[2] (see Methods).

Data was used in the Figure 1b-e, Supplementary figures: S2 and S8a-b.

Torre study (UMI).

Single-cell RNA-seq expression data were downloaded from GEO GSE99330 (GSE99330_dropseqUPM.txt.gz)[12]. This data matrix was converted to counts using a code kindly provided by the author of SAVER[9].

There are 32287 genes and 8640 cells in the raw data. First cells with less than 2000 genes detected or where the gene "GAPDH" could not be detected were filtered out. Second, genes with mean expression less or equal to 0.01 were removed. Third, the gene "GAPDH" was removed because it is used as a proxy for cell size[9] by normalizing it to the mean β which was estimated using smFISH data (see Methods). The final filtered dataset contained 9289 genes and 1134 cells. 5 samples were generated to form 3D array in bayNorm.

For smFISH data, we filtered out cells with "GAPDH" counts below the bottom 10th percentile or above the top 10th percentile of 'GAPDH" counts. smFISH data were then normalized by the expression of GAPDH divided by GAPDH mean expression[9].

We fitted a linear regression of the mean expression of filtered dataset on that of the normalized smFISH datset (Supplementary Figure S11c). The coefficient of explanatory variable was then used as $\bar{\beta}$. "GAPDH" expression, which was filtered out previously, was divided by the median and multiplied by $\bar{\beta}$ (see Methods).

Data was used in the Figure 2b,d,f,h, Figure 3a-b, Supplementary figures: S6, S8e-f, S10a and S26.

Soumillon study (UMI).

The dataset was downloaded from GEO GSE53638 (GSE53638_D3_UMI.dat.gz for the single cell data and GSE53638_D3_Bulk_UMI.dat.gz for the bulk data)[20]. DE detection was performed between the stage-3 differentiated cells at day 0 (D3T0) and day 7 (D3T7) (23895 genes and 1949 cells) [18]. Cells with library sizes below the bottom and above the top 5th percentiles were filtered out. Genes with mean expression across two groups greater than 0.05 were retained, resulting in a dataset of 1754 cells with 8586 genes (832 cells and 922 cells belonging to day 0 and day 7 time-points respectively). Using the same 8586 genes in the bulk dataset, the reference DE genes were defined to be the top 1000 genes which have the greatest log fold-change in the corresponding bulk RNA-seq data[18]. 10 samples were generated to form 3D array in bayNorm.

Based on the smFISH data, $\bar{\beta}$ is expected to be in the range of $1 - 2\%$[20]. Here we set $\bar{\beta} = 2\%$. Scaling factors were estimated using R package scran[6], and then normalized to 0.02 (see Methods).

Data was used in the Figure 3d and Supplementary figures: S16.

Zeisel study (UMI).

The dataset was downloaded from GEO GSE60361 [21]. We followed the filtering criterion adapted from [1] to obtain the final dataset with 9986 genes and 3005 cells. For bayNorm, β was estimated using default setting in bayNorm. The same β was used for Scaling method. One sample of 3D array output from bayNorm was used in analysis.

Data was used in the Figure 4d-e.

Baron study (UMI).

The preprocessed data[22] was downloaded based on the guidline provided in https://github.com/mohuangx/SAVER-paper (baron_human_ref.rds) [9], where there are 2284 genes and 1076 cells. The detailed filtering criterion can be found in [9]. For bayNorm, β was estimated using default setting in bayNorm. The same β was used for Scaling method. One sample of 3D array output from bayNorm was used in analysis. Seurat[23] was used for clustering and assigning cell labels on scaling and bayNorm normalized data independently.

Data was used in the Figure S24-25.

Chen study (UMI).

The preprocessed data[24] was downloaded based on the guidline provided in https://github.com/mohuangx/SAVER-paper (chen_ref.rds) [9], where there are 2159 genes and 7712 cells. The detailed filtering criterion can be found in [9]. For bayNorm, β was estimated using default setting in bayNorm. The same β was used for Scaling method. One sample of 3D array output from bayNorm was used in analysis.

Data was used in the Figure S24-25.

Saint study (UMI).

bayNorm normalized data of 2000 Homogeneous fission yeasts with 1011 genes were used (See [25] for more details).

Data was used in the Figure 3a-b.

Supplementary Note 5: Normalization methods and relevant R packages

Splatter, R package version 1.4.1

For both UMI and non-UMI data, the default settings of Splatter were used for estimating parameters from the input data and simulating scRNAseq data.

For non-UMI data in Supplementary Figure S7, H1_P24 single cell data were divided by 20 and then rounded as an input for Splatter.

SAVER, R package version 1.1.1

We used the default settings for SAVER throughout the paper. When estimating mean, CV and Gini coefficients, for both bayNorm and SAVER we generated 5 samples for the Torre study and 20 samples the Grün study (3D arrays). The mean, CV and Gini were estimated across the cells and samples. In the 6 simulation studies, 10 samples were generated from posterior for both bayNorm and SAVER. In the Klein (Figure 3a-b), Tung and Soumillon studies, 5 samples were generated. In Tung study, SAVER was applied within each individual.

SCnorm, R package version 1.1.0

We used the default settings in SCnorm except for UMI datasets and simulated data, where "dither-Counts=TRUE" was used as UMI data contains tied counts.

scImpute, R package version 0.0.6

We applied scImpute using its default settings. In the Tung study, scImpute was applied on each individual independently.

MAGIC, R package version 0.1.0

MAGIC was applied using its default settings. In the Tung study, MAGIC was applied within each individual.

DCA, Python package version 0.2.2

DCA was applied using its default settings. In Tung study, since genes with 0 counts across cells within each individual could be filtered out, we applied DCA across all cells.

Scaling method

Throughout the paper, the scaling method refers to $\tilde{x}_{ij} = \frac{x_{ij}}{\beta_j}$. In UMI datasets and simulated data, β used in scaling method are as the same as that used in bayNorm.

Seurat, R package version 2.3.4

Seurat[23] was used in Baron and Chen studies for clustering and assigning cell labels on scaling and bayNorm normalized data independently.

BASiCS, R package version 1.4.7

BASiCS[7, 27] was applied on the Group 2 of SIM DE study II (Fig S28) with parameter setting: Thin=10, Burn=10000 and N=20000. The rest were set as default.

DESeq2, R package version 1.22.2

DESeq2[4] was applied on the Group 2 of SIM DE study II (Fig S28) with default setting.

Code availability

The R package bayNorm is available at https://github.com/WT215/bayNorm.

The codes for producing figures in the paper are provided at https://github.com/WT215/bayNorm_papercode.

In the Bacher study, the code for running MAST and log fold change calculation was kindly provided by Rhonda Bacher, the author of SCnorm ([15]).

In the Torre study, the code for transforming counts per million normalized data to UMI data was kindly provided by Mo Huang, the author of SAVER ([9]).

Algorithm 1 Pseudo code for bayNorm

```
Given the raw data $x_{ij}$ and the estimated capture efficiencies $\beta_j$ do the following:
Simple normalization
for $i \in \{1, \dots, P\}$ do
    for $j \in \{1, \dots, Q\}$ do
        $x_{ij}^s = x_{ij}/\beta_j$
    end for
end for
MME estimation
for $i \in \{1, \dots, P\}$ do
    estimate MME size $\mu_i$ and $\phi_i$
end for
BB estimation
for $i \in \{1, \dots, P\}$ do
    estimate BB size $\phi'_i$
end for
Adjust MME size
for $i \in \{1, \dots, P\}$ do
    Adjust $\phi_i$ according to $\phi'_i$
end for
Generate normalized data
for $i \in \{1, \dots, P\}$ do
    for $j \in \{1, \dots, Q\}$ do
        Draw samples/mean/mode from posterior distribution of $x_{ij}^0$
    end for
end for
```

References

[1] Catalina A Vallejos, Davide Risso, Antonio Scialdone, Sandrine Dudoit, and John C Marioni. Normalizing single-cell rna sequencing data: challenges and opportunities. *Nature methods*, 14 (6):565, 2017.

[2] Allon M Klein, Linas Mazutis, Ilke Akartuna, Naren Tallapragada, Adrian Veres, Victor Li, Leonid Peshkin, David A Weitz, and Marc W Kirschner. Droplet barcoding for single-cell transcriptomics applied to embryonic stem cells. *Cell*, 161(5):1187–1201, 2015.

[3] Dominic Grün, Lennart Kester, and Alexander Van Oudenaarden. Validation of noise models for single-cell transcriptomics. *Nature methods*, 11(6):637–640, 2014.

[4] Michael I Love, Wolfgang Huber, and Simon Anders. Moderated estimation of fold change and dispersion for rna-seq data with deseq2. *Genome Biol.*, 15(12):550, 2014.

[5] Mark D Robinson and Alicia Oshlack. A scaling normalization method for differential expression analysis of rna-seq data. *Genome biology*, 11(3):R25, 2010.

[6] Aaron TL Lun, Karsten Bach, and John C Marioni. Pooling across cells to normalize single-cell rna sequencing data with many zero counts. *Genome biology*, 17(1):75, 2016.

[7] Catalina A Vallejos, John C Marioni, and Sylvia Richardson. Basics: Bayesian analysis of single-cell sequencing data. *PLoS computational biology*, 11(6):e1004333, 2015.

[8] Evan Z Macosko, Anindita Basu, Rahul Satija, James Nemesh, Karthik Shekhar, Melissa Goldman, Itay Tirosh, Allison R Bialas, Nolan Kamitaki, Emily M Martersteck, et al. Highly parallel genome-wide expression profiling of individual cells using nanoliter droplets. *Cell*, 161 (5):1202–1214, 2015.

[9] Mo Huang, Jingshu Wang, Eduardo Torre, Hannah Dueck, Sydney Shaffer, Roberto Bonasio, John I Murray, Arjun Raj, Mingyao Li, and Nancy R Zhang. Saver: gene expression recovery for single-cell rna sequencing. *Nature Methods*, page 1, 2018.

[10] Luke Zappia, Belinda Phipson, and Alicia Oshlack. Splatter: simulation of single-cell rna sequencing data. *Genome biology*, 18(1):174, 2017.

[11] Florian Wagner, Yun Yan, and Itai Yanai. K-nearest neighbor smoothing for high-throughput single-cell rna-seq data. *bioRxiv*, page 217737, 2018.

[12] Eduardo Torre, Hannah Dueck, Sydney Shaffer, Janko Gospocic, Rohit Gupte, Roberto Bonasio, Junhyong Kim, John Murray, and Arjun Raj. Rare cell detection by single-cell rna sequencing as guided by single-molecule rna fish. *Cell systems*, 6(2):171–179, 2018.

[13] Po-Yuan Tung, John D Blischak, Chiaowen Joyce Hsiao, David A Knowles, Jonathan E Burnett, Jonathan K Pritchard, and Yoav Gilad. Batch effects and the effective design of single-cell gene expression studies. *Scientific reports*, 7:39921, 2017.

[14] Bradley P Carlin and Thomas A Louis. *Bayesian methods for data analysis*. CRC Press, 2008.

[15] Rhonda Bacher, Li-Fang Chu, Ning Leng, Audrey P Gasch, James A Thomson, Ron M Stewart, Michael Newton, and Christina Kendziorski. Scnorm: robust normalization of single-cell rna-seq data. *Nature methods*, 14(6):584, 2017.

[16] Maria K Jaakkola, Fatemeh Seyednasrollah, Arfa Mehmood, and Laura L Elo. Comparison of methods to detect differentially expressed genes between single-cell populations. *Briefings in bioinformatics*, 18(5):735–743, 2016.

[17] Anoop P Patel, Itay Tirosh, John J Trombetta, Alex K Shalek, Shawn M Gillespie, Hiroaki Wakimoto, Daniel P Cahill, Brian V Nahed, William T Curry, Robert L Martuza, et al. Single-cell rna-seq highlights intratumoral heterogeneity in primary glioblastoma. *Science*, 344(6190): 1396–1401, 2014.

[18] Chengzhong Ye, Terence P Speed, and Agus Salim. Decent: Differential expression with capture efficiency adjustment for single-cell rna-seq data. *bioRxiv*, page 225177, 2017.

[19] Catalina A Vallejos, Sylvia Richardson, and John C Marioni. Beyond comparisons of means: understanding changes in gene expression at the single-cell level. *Genome biology*, 17(1):70, 2016.

[20] Magali Soumillon, Davide Cacchiarelli, Stefan Semrau, Alexander van Oudenaarden, and Tarjei S Mikkelsen. Characterization of directed differentiation by high-throughput single-cell rna-seq. *BioRxiv*, page 003236, 2014.

[21] Amit Zeisel, Ana B Muñoz-Manchado, Simone Codeluppi, Peter Lönnerberg, Gioele La Manno, Anna Juréus, Sueli Marques, Hermany Munguba, Liqun He, Christer Betsholtz, et al. Cell types in the mouse cortex and hippocampus revealed by single-cell rna-seq. *Science*, 347(6226):1138–1142, 2015.

[22] Maayan Baron, Adrian Veres, Samuel L Wolock, Aubrey L Faust, Renaud Gaujoux, Amedeo Vetere, Jennifer Hyoje Ryu, Bridget K Wagner, Shai S Shen-Orr, Allon M Klein, et al. A single-cell transcriptomic map of the human and mouse pancreas reveals inter-and intra-cell population structure. *Cell systems*, 3(4):346–360, 2016.

[23] Andrew Butler, Paul Hoffman, Peter Smibert, Efthymia Papalexi, and Rahul Satija. Integrating single-cell transcriptomic data across different conditions, technologies, and species. *Nature biotechnology*, 36(5):411, 2018.

[24] Renchao Chen, Xiaoji Wu, Lan Jiang, and Yi Zhang. Single-cell rna-seq reveals hypothalamic cell diversity. *Cell reports*, 18(13):3227–3241, 2017.

[25] Malika Saint, François Bertaux, Wenhao Tang, Xi-Ming Sun, Laurence Game, Anna Köferle, Jürg Bähler, Vahid Shahrezaei, and Samuel Marguerat. Single-cell imaging and rna sequencing reveal patterns of gene expression heterogeneity during fission yeast growth and adaptation. *Nature microbiology*, 4(3):480–491, 2019.

[26] Stephanie C Hicks, F William Townes, Mingxiang Teng, and Rafael A Irizarry. Missing data and technical variability in single-cell rna-sequencing experiments. *Biostatistics*, 19(4):562–578, 2017.

[27] Nils Eling, Arianne C Richard, Sylvia Richardson, John C Marioni, and Catalina A Vallejos. Correcting the mean-variance dependency for differential variability testing using single-cell rna sequencing data. *Cell systems*, 7(3):284–294, 2018.

Supplementary figures

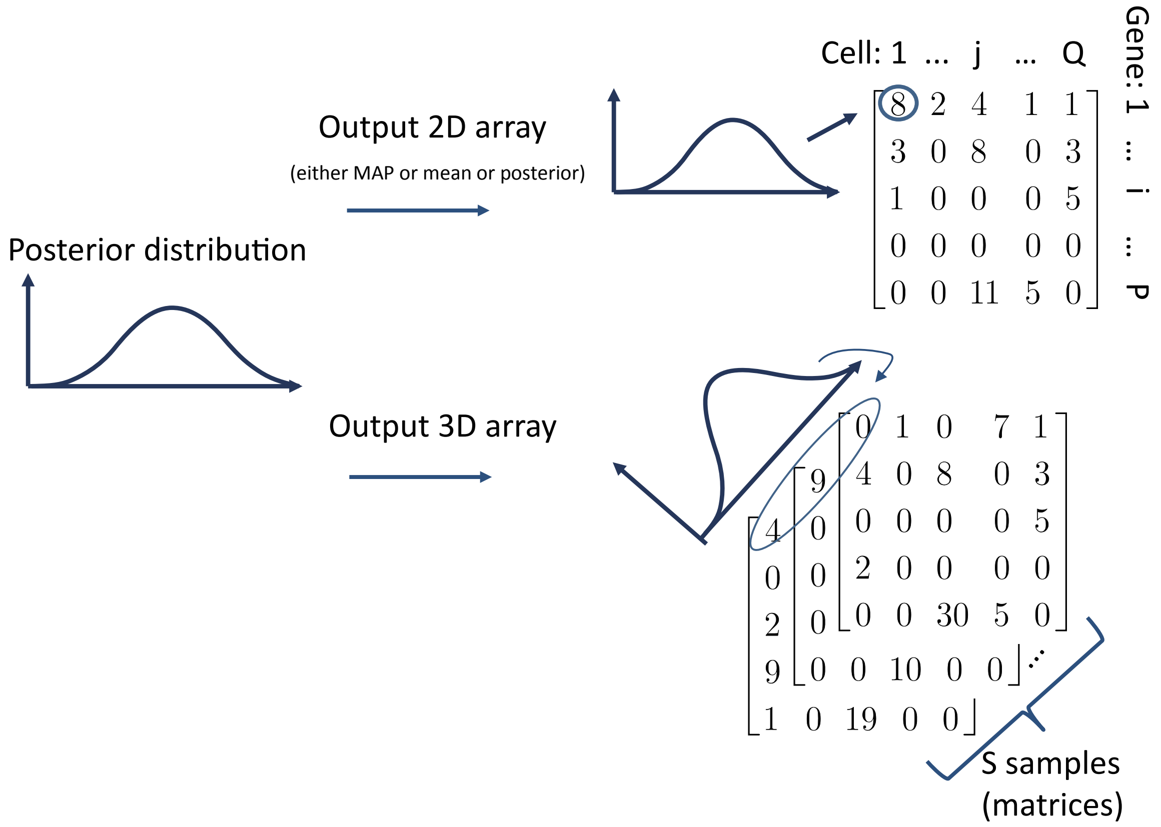

Figure S1: Output of bayNorm. For each gene in each cell, we have a posterior distribution as bayNorm is a Baysian method (See methods). Final bayNorm output is either S samples randomly sampled from the posterior distributions (3D arrays), or the mode or mean of the posterior used as point estimates (2D arrays).

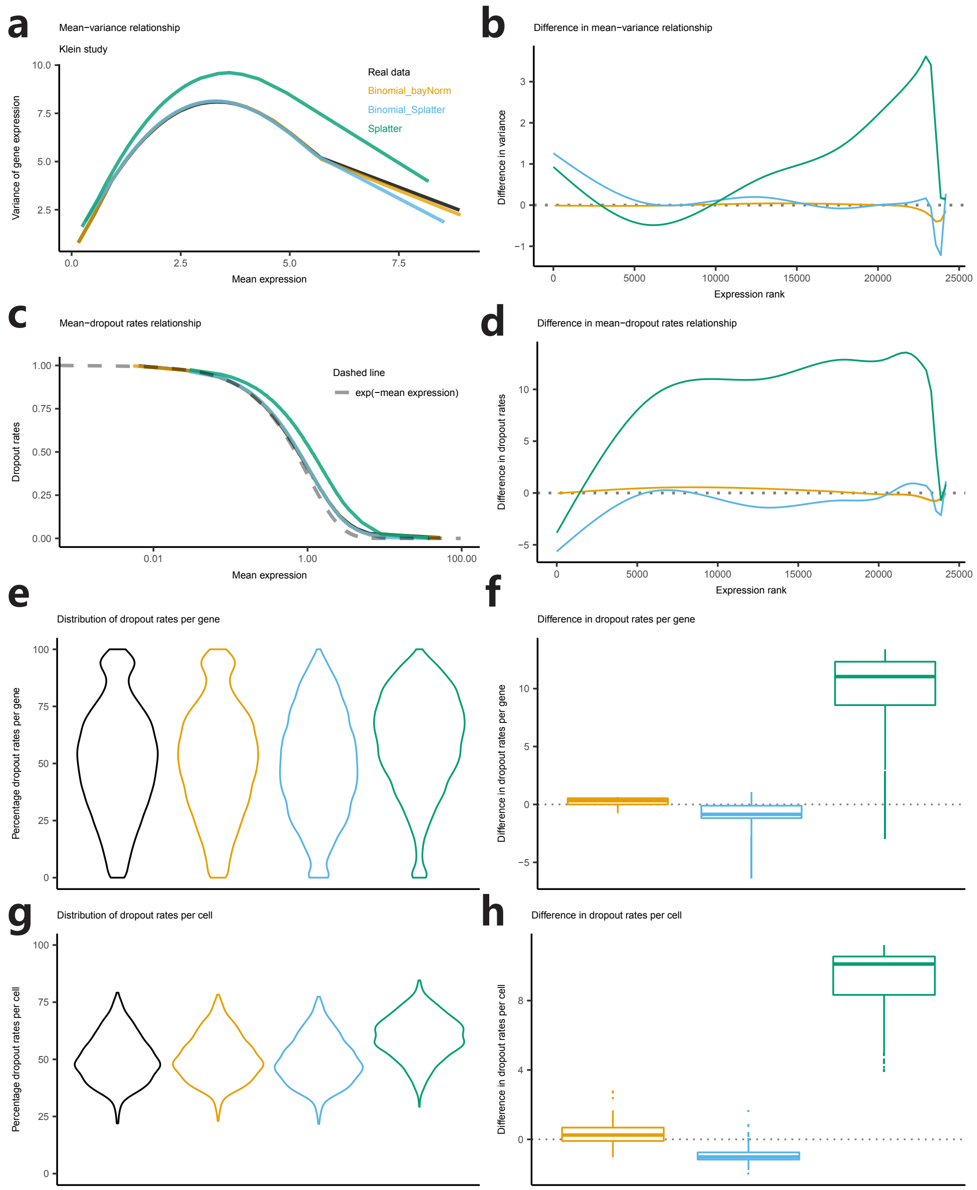

Figure S2: Simulation analysis based on the Klein study. Comparison between simulated data and experimental data in terms of: (a-b) variance-mean relationship, (c-d) dropout rates-mean relationship, (e-f) distribution of proportions of zeros per gene, (g-h) distribution of proportions zeros per cell. (b), (d), (f) and (h): ranked difference between statistics of experimental data and simulated data for (a), (c), (e) and (g) respectively. The smoothed lines in (a) and (c) were obtained by binning x values and calculating the mean of y values in each bin[26]. The smoothed lines in (b) and (d) were generated by ggplot2 (geom_smooth with method set to “auto”).

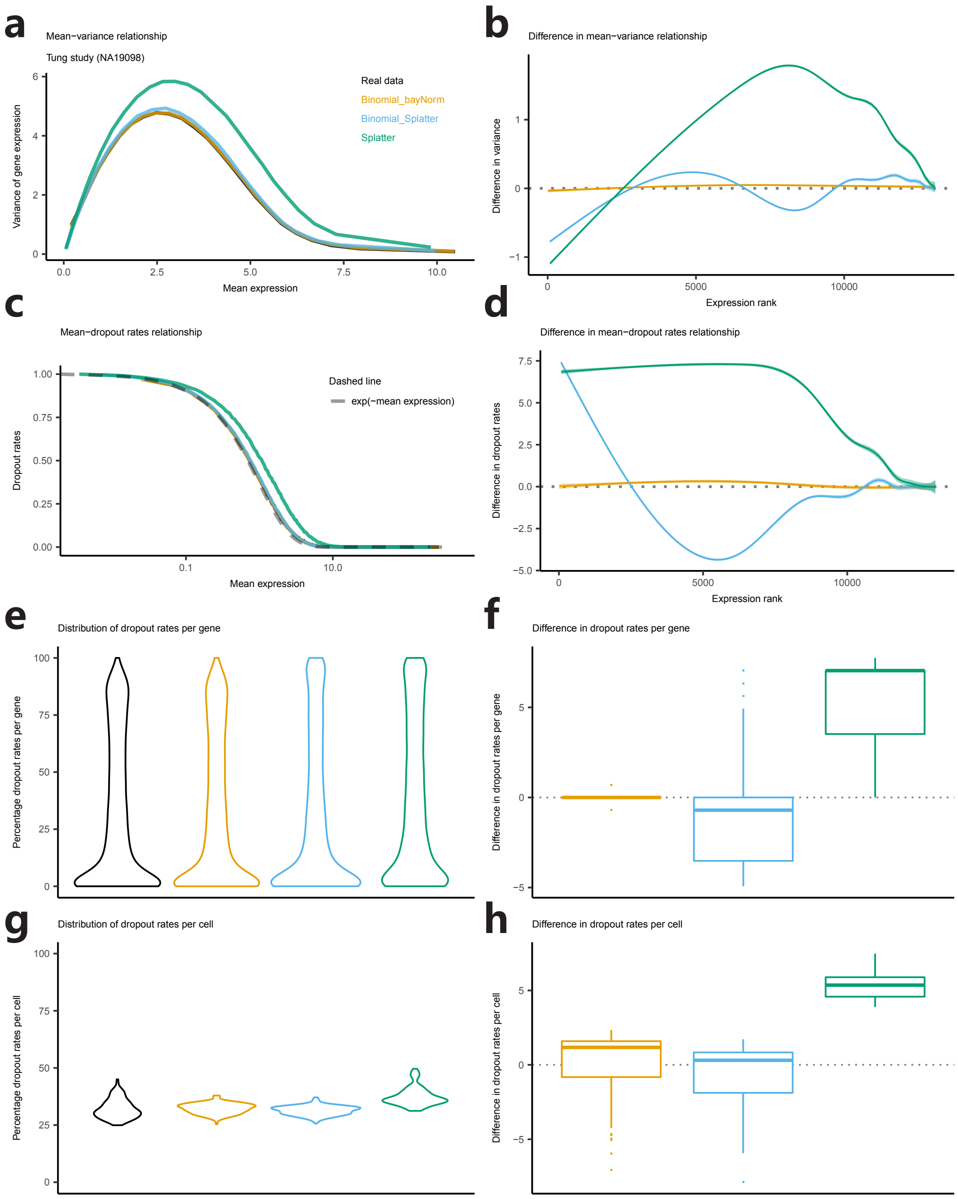

Figure S3: Simulation analysis based on the Tung study (Individual NA19098). Comparison between simulated data and real data in terms of: (a-b) variance-mean relationship, (c-d) dropout rates-mean relationship, (e-f) distribution of proportions of zeros per gene, (g-h) distribution of proportions zeros per cell. (b), (d), (f) and (h): ranked difference between statistics of experimental data and simulated data for (a), (c), (e) and (g) respectively. The smoothed lines in (a) and (c) were obtained by binning x values and calculating the mean of y values in each bin[26]. The smoothed lines in (b) and (d) were generated by ggplot2 (geom_smooth with method set to "auto").

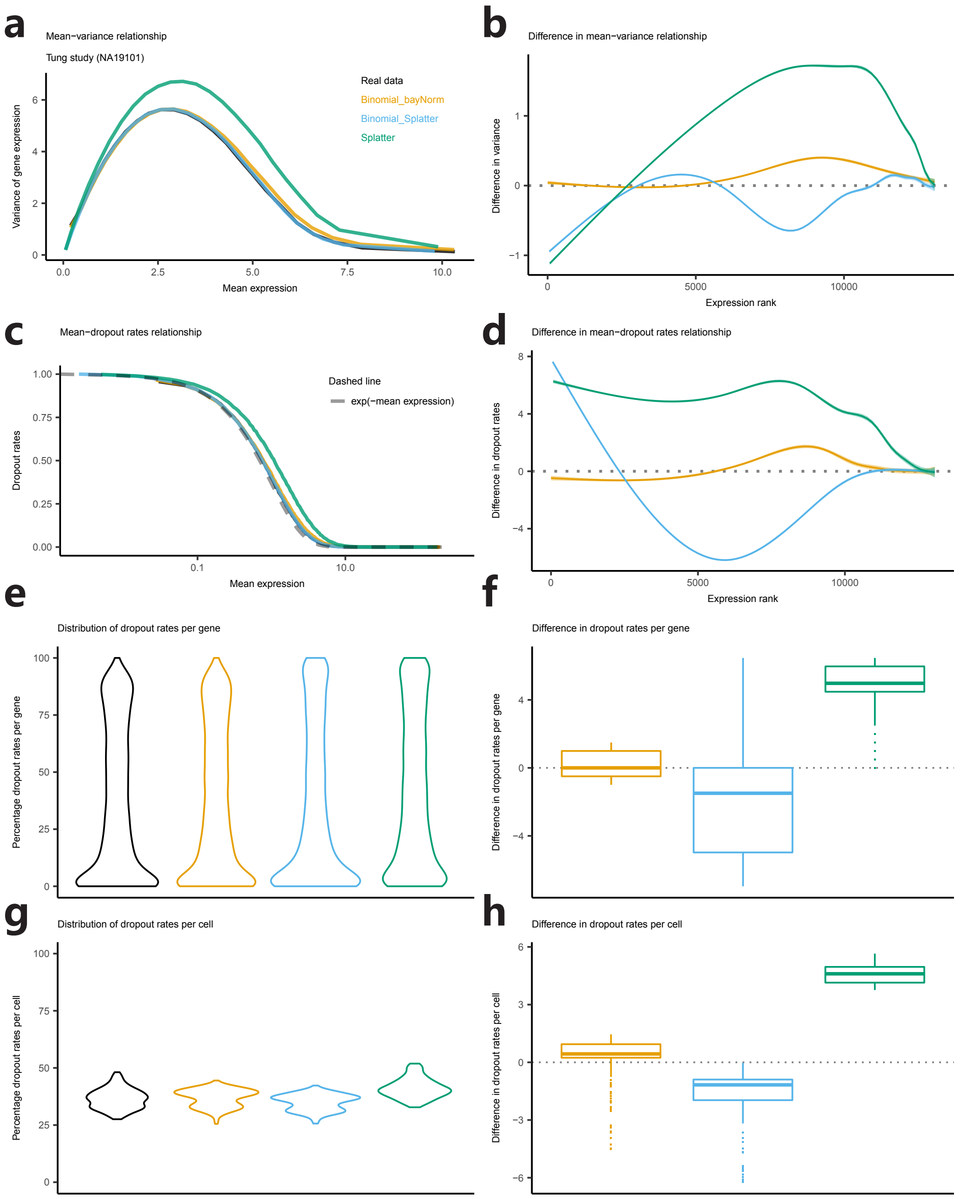

Figure S4: Simulation analysis based on the Tung study (Individual NA19101). Comparison between simulated data and real data in terms of: (a-b) variance-mean relationship, (c-d) dropout rates-mean relationship, (e-f) distribution of proportions of zeros per gene, (g-h) distribution of proportions zeros per cell. (b), (d), (f) and (h): ranked difference between statistics of experimental data and simulated data for (a), (c), (e) and (g) respectively. The smoothed lines in (a) and (c) were obtained by binning x values and calculating the mean of y values in each bin[26]. The smoothed lines in (b) and (d) were generated by ggplot2 (geom_smooth with method set to "auto").

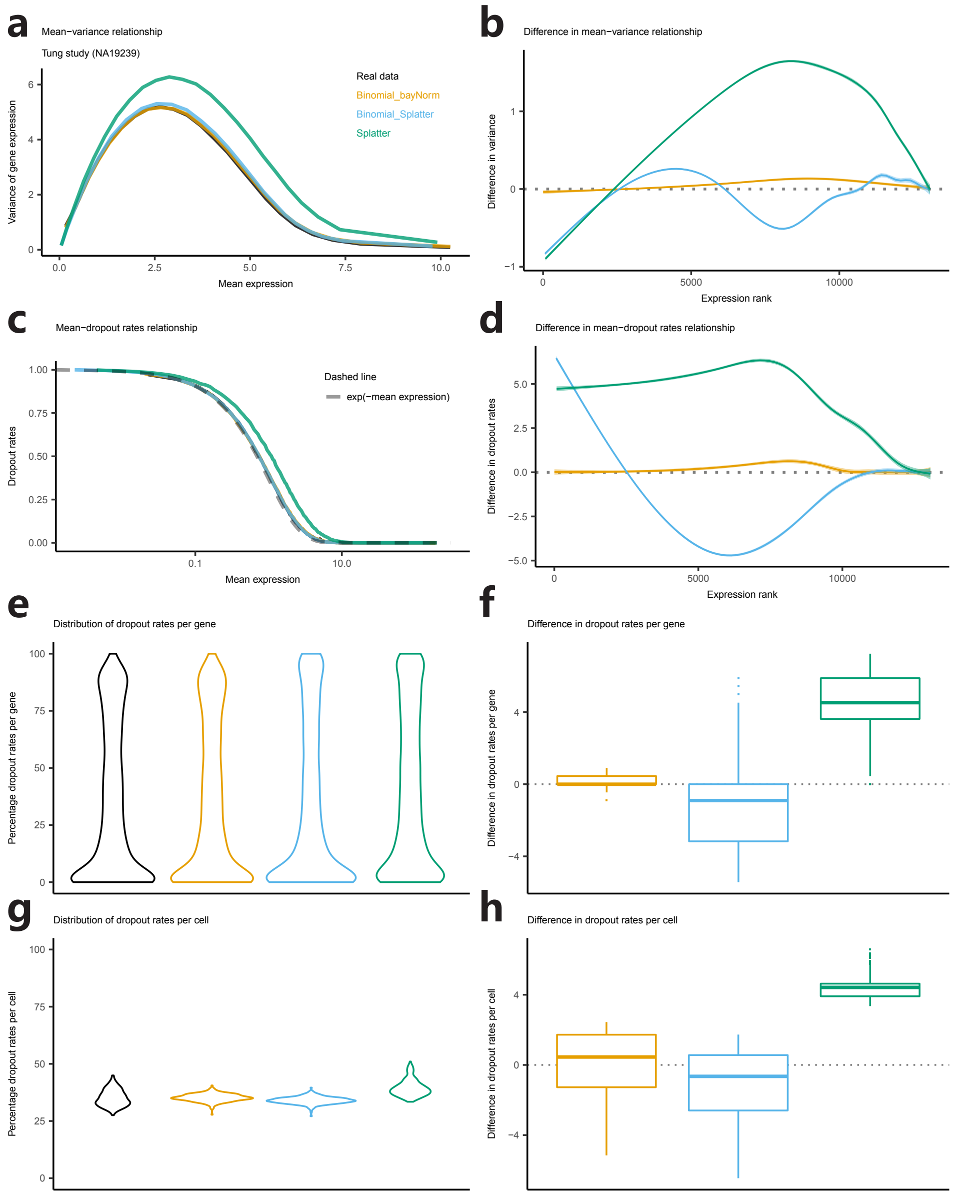

Figure S5: Simulation analysis based on the Tung study (Individual NA19239). Comparison between simulated data and real data in terms of: (a-b) variance-mean relationship, (c-d) dropout rates-Mean relationship, (e-f) distribution of proportions of zeros per gene, (g-h) distribution of proportions zeros per cell. (b), (d), (f) and (h): ranked difference between statistics of experimental data and simulated data for (a), (c), (e) and (g) respectively. The smoothed lines in (a) and (c) were obtained by binning x values and calculating the mean of y values in each bin[26]. The smoothed lines in (b) and (d) were generated by ggplot2 (geom_smooth with method set to "auto").

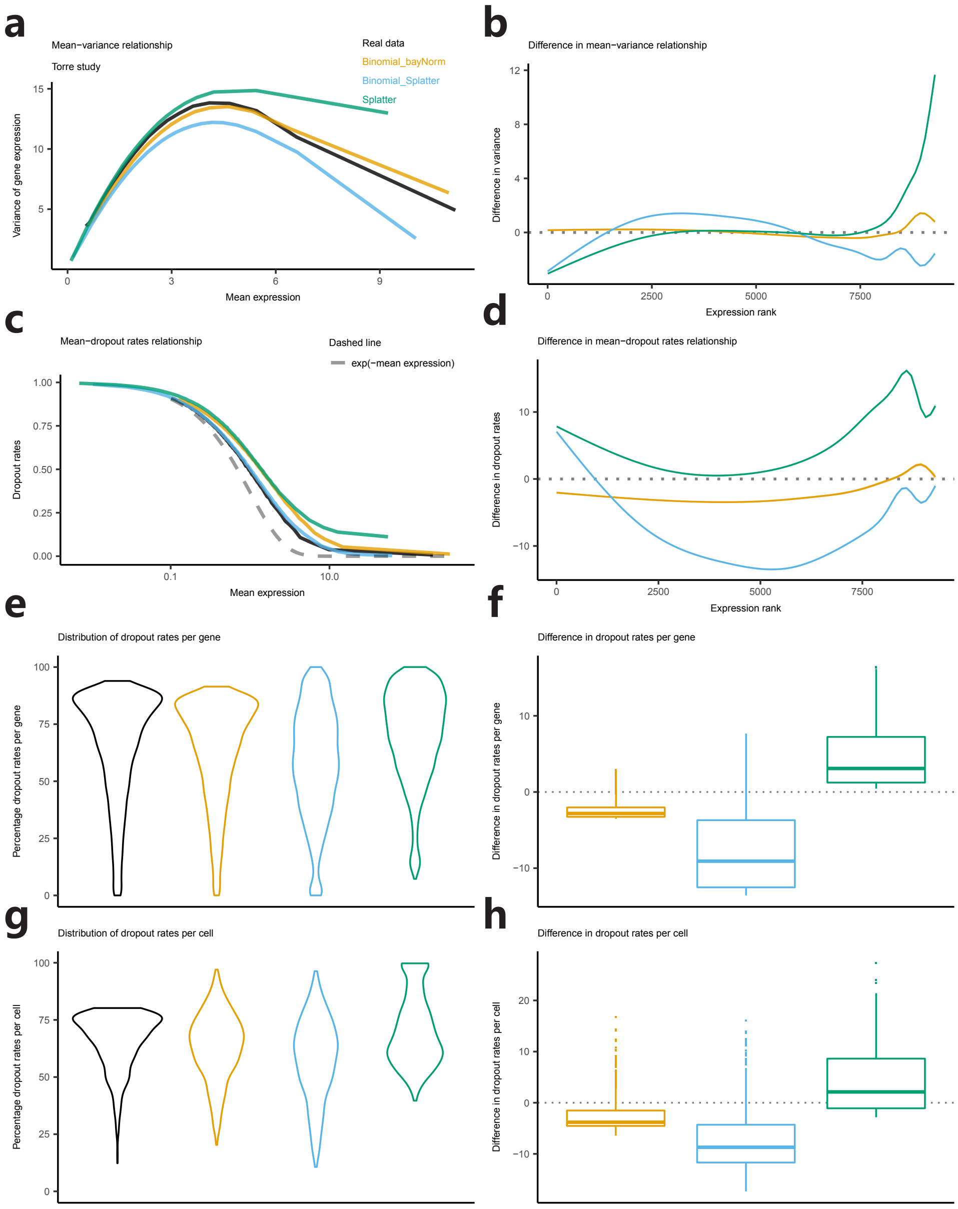

Figure S6: Simulation analysis based on the Torre study. Comparison between simulated data and real data in terms of: (a-b) variance-mean relationship, (c-d) dropout rates-mean relationship, (e-f) distribution of proportions of zeros per gene, (g-h) distribution of proportions zeros per cell. (b), (d), (f) and (h): ranked difference between statistics of experimental data and simulated data for (a), (c), (e) and (g) respectively. The smoothed lines in (a) and (c) were obtained by binning x values and calculating the mean of y values in each bin[26]. The smoothed lines in (b) and (d) were generated by ggplot2 (geom_smooth with method set to "auto").

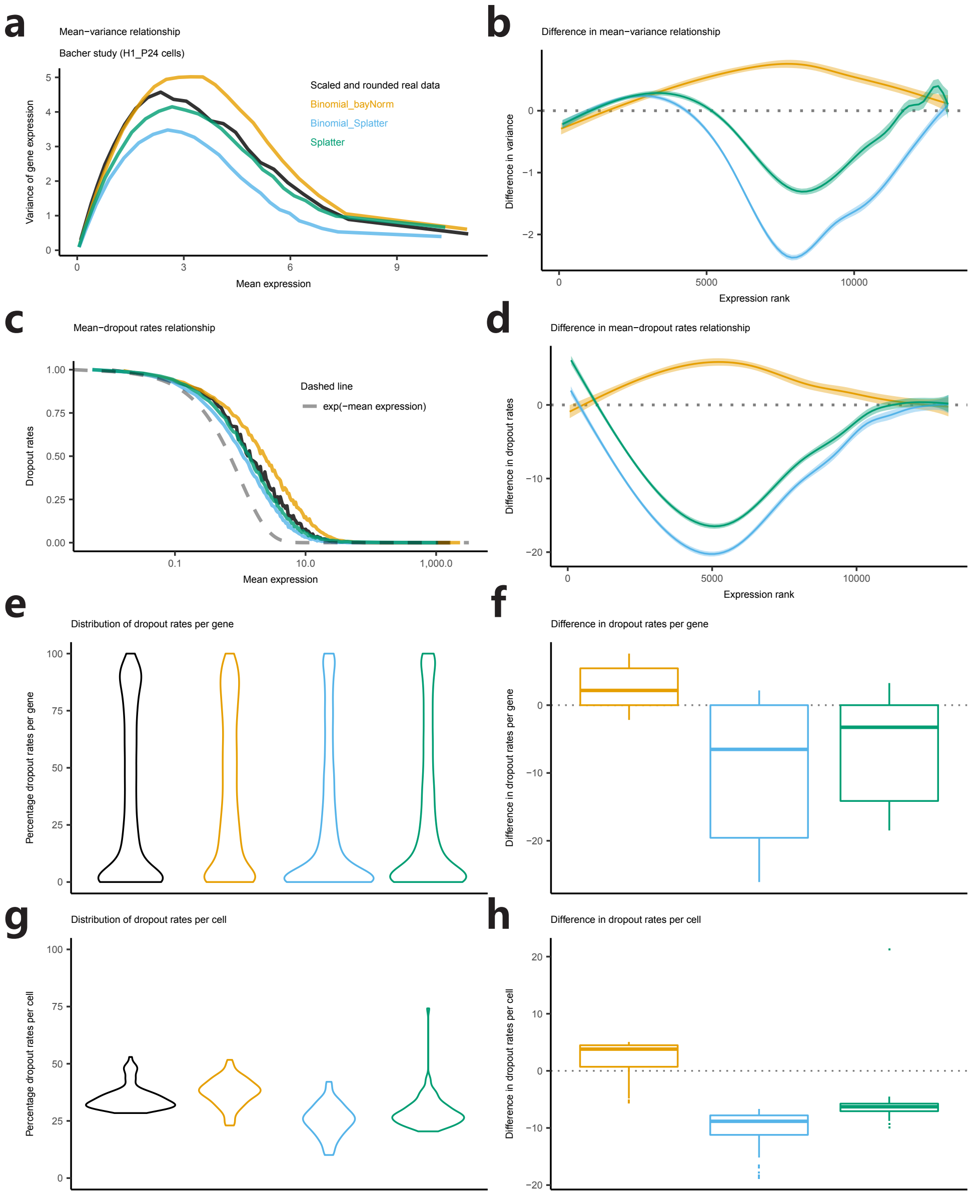

Figure S7: Simulation analysis based on H1_P24 cells in the Bacher study. Comparison between simulated data and real data in terms of: (a-b) variance-mean relationship, (c-d) dropout rates-mean relationship, (e-f) distribution of proportions of zeros per gene, (g-h) distribution of proportions zeros per cell. (b), (d), (f) and (h): ranked difference between statistics of experimental data and simulated data for (a), (c), (e) and (g) respectively. The smoothed lines in (a) and (c) were obtained by binning x values and calculating the mean of y values in each bin33. The smoothed lines in (b) and (d) were generated by ggplot2 (geom_smooth with method set to "auto"). Experimental data were scaled by 20 and rounded before being used as input of the three simulation protocols.

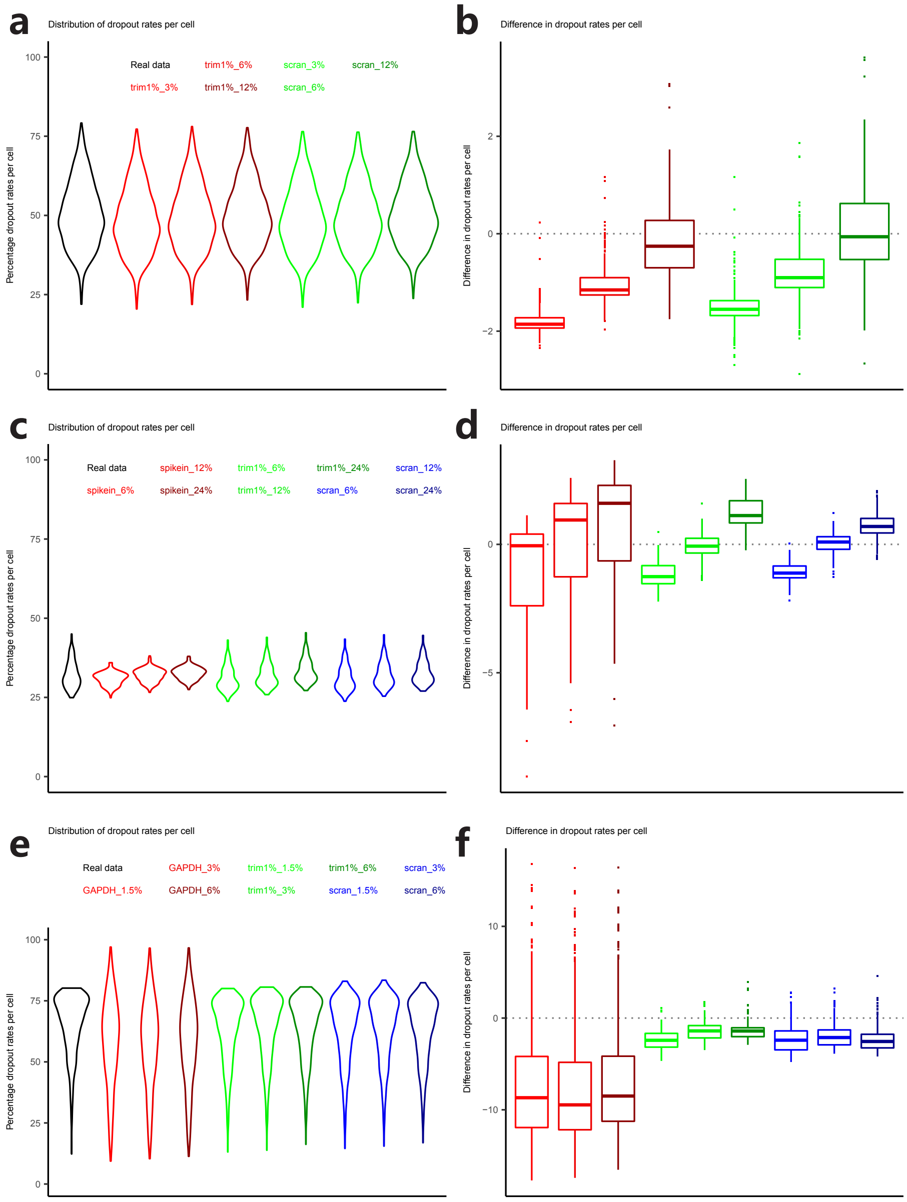

Figure S8: Impact of different mean capture efficiencies and different size factor estimates on the "Binomial_Splatter" simulation protocol. Results are based on (a-b) the Klein study. (c-d) the NA19098 sample from the Tung study. (e-f) the Torre study. Scaling factors were estimated using different methods: (1) "trim1%": 1% of counts were trimmed from each end of the counts in a specific cell before computing the mean. (2): "scran": scaling factors were estimated with the R package scran[6]. (3) "spikein": total counts of observed spike-ins in each cell were used as scaling factors. (4) "GAPDH": the expression of the housekeeping gene GAPDH was used as scaling factors. The percentage at the end of each label indicates the mean capture efficiency $\bar{\beta}$ (see Methods).

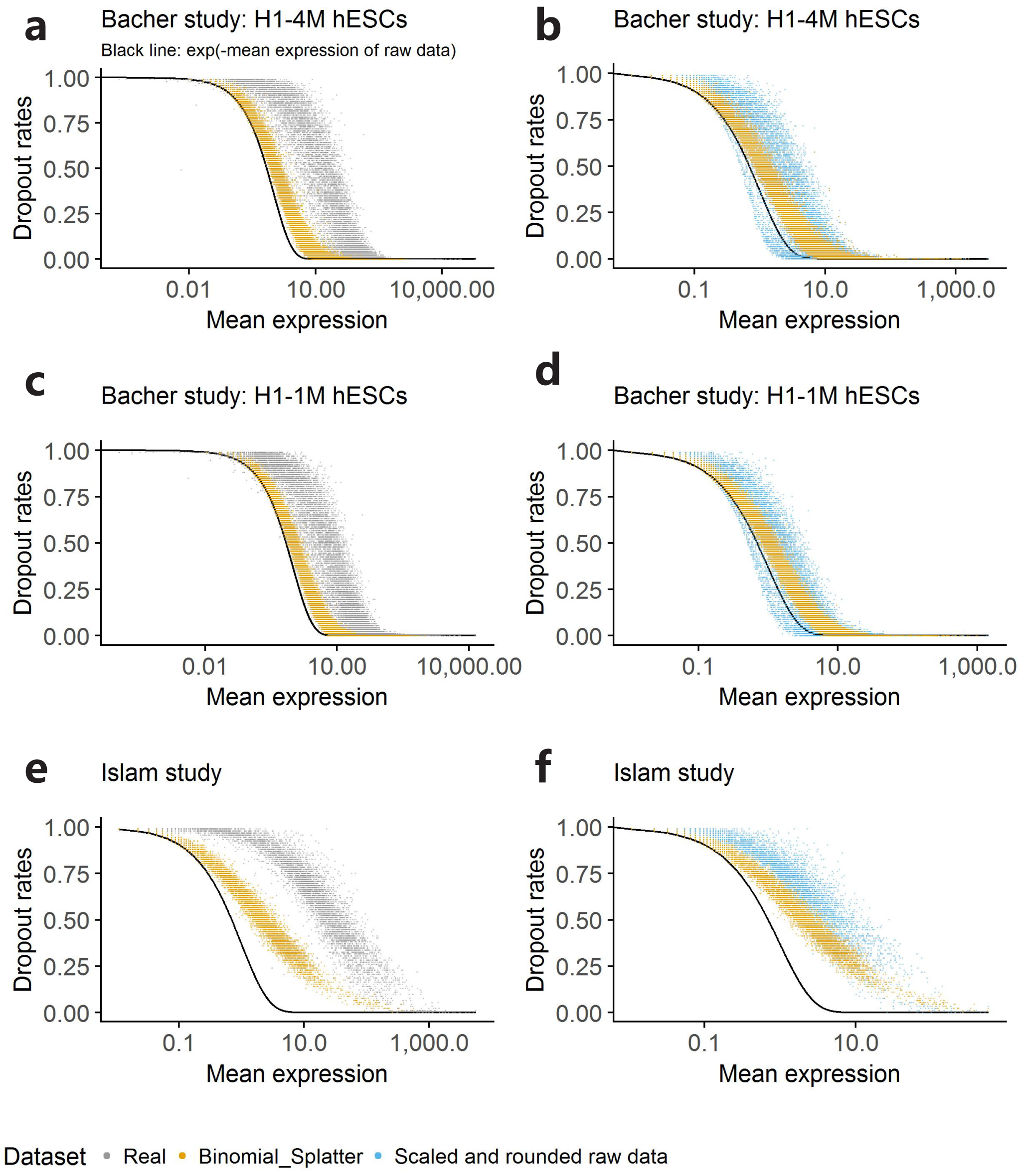

Figure S9: Comparison between simulated data and raw experimental data in terms of the relationship between dropout rates and mean expression. (a-b) non-UMI data from the Bacher study (H1 hESCs from the 4 million mapped reads group). In (b) raw experimental data were divided by 20 and rounded. (c-d) non-UMI data from the Bacher study (H1 hESCs from the 1 million mapped reads group). In (d) raw experimental data were divided by 10 and rounded. (e-f) non-UMI data from the Islam study. In (f) raw experimental data were divided by 10 and rounded. (b), (d) and (f) are comparisons between Binomial_Splatter simulated data and scaled and rounded real experimental data. Parameters in Binomial_Splatter simulations were generated from scaled raw data as illustrated in the Supplementary Information.

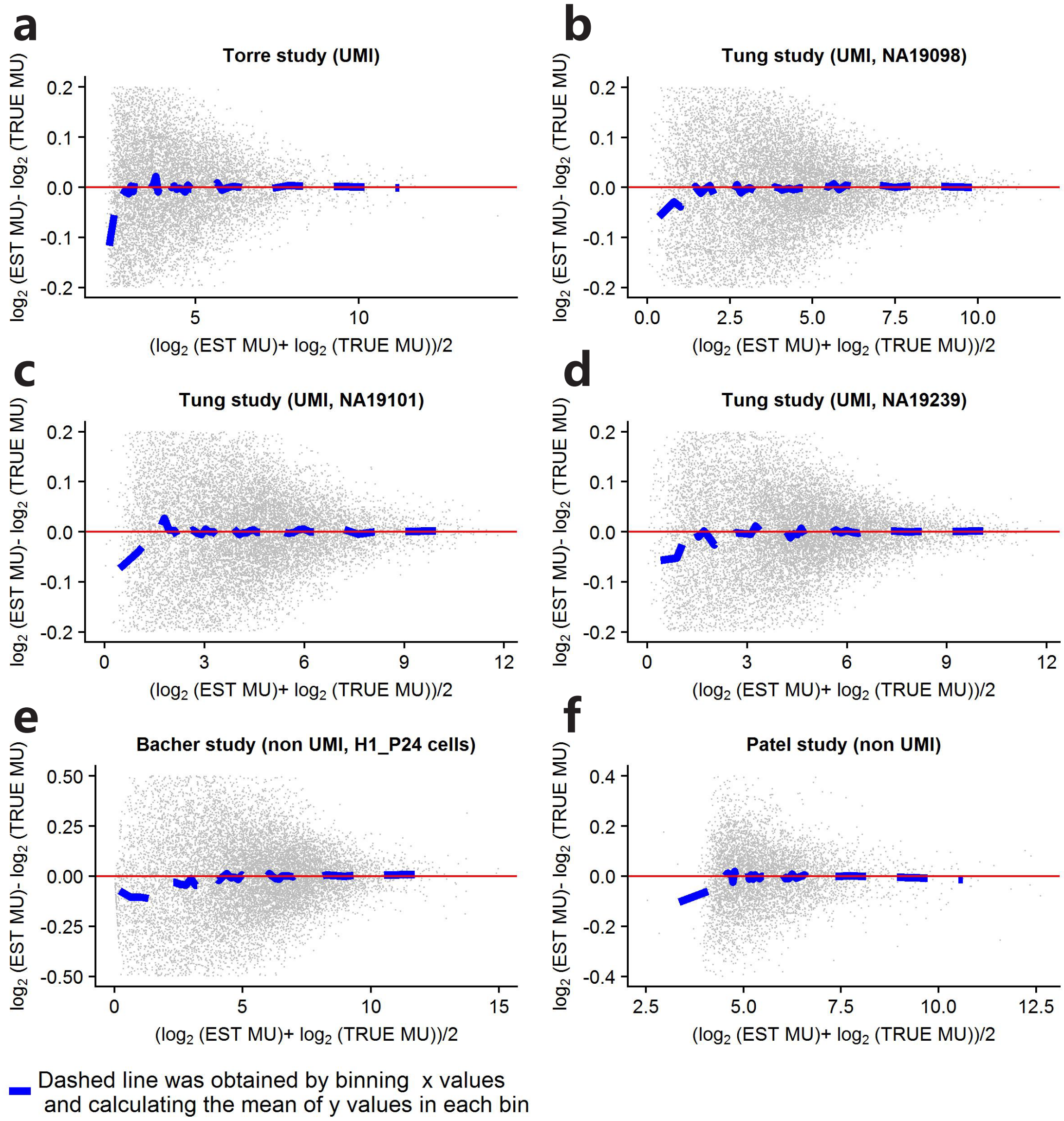

Figure S10: MA plots based on simulated data using the Binomial_bayNorm protocol. "TRUE MU" stands for the MME estimated μ output from bayNorm and "EST MU" stands for the mean expression of Binomial_bayNorm simulated data scaled by the β used in bayNorm. Binomial_bayNorm simulation protocol was applied to (a) the Torre study, (b) individual NA19098 from the Tung study, (c) individual NA19101, (d) individual NA19239, (e) the Bacher study and (f) the Patel study. The dashed line was obtained by binning x values and calculating the mean of y values in each bin[26].

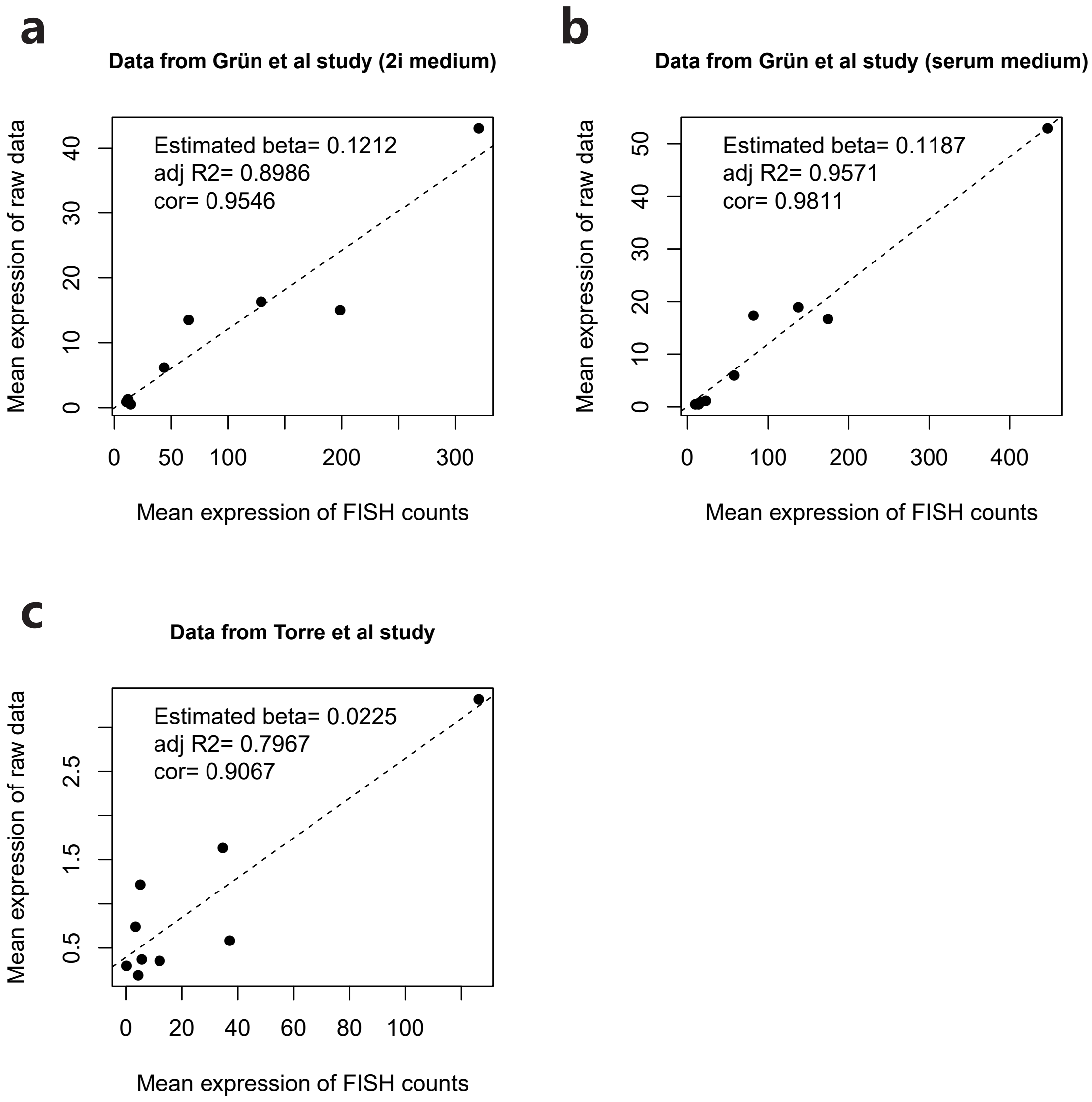

Figure S11: Linear regression of mean expression of scRNA-seq experimental raw data vs smFISH data. (a) 2i medium single cell data from the Grün study. (b) Serum medium single cell data from the Grün study. (c) Data from the Torre study. The coefficient of explanatory variable of linear regression is used as mean beta.

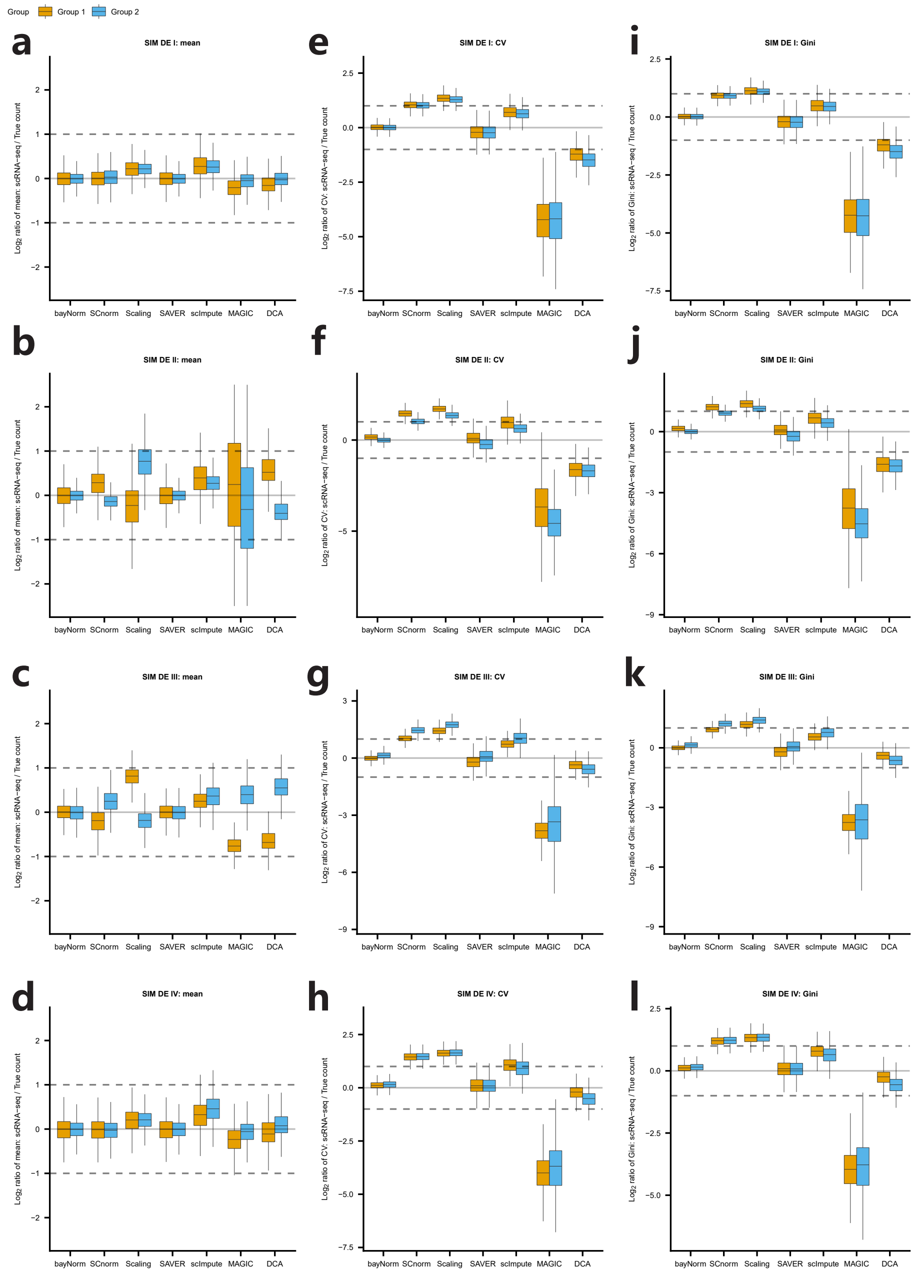

Figure S12: Recovering the mean, CV and Gini of gene expression using simulated scRNA-seq data. For the four simulation studies: SIM DE I-IV (see Supplementary Information for details about simulation studies), Log_2 ratio between true simulated data (dataset before binomial downsampling) and normalized simulated scRNA-seq data for mean gene expression (a-d), CV (e-h) and Gini coefficients (i-l). (a), (e) and (i) are based on SIM DE I simulated data. (b), (f) and (j) are based on SIM DE II simulated data. (c), (g) and (k) are based on SIM DE III simulated data. (d), (h) and (l) are based on SIM DE IV simulated data. Except for the bayNorm and scaling methods, the normalized datasets have been divided by their corresponding mean capture efficiencies (either 0.1 or 0.05) for a fair comparison. For bayNorm and SAVER, 3D arrays were used.

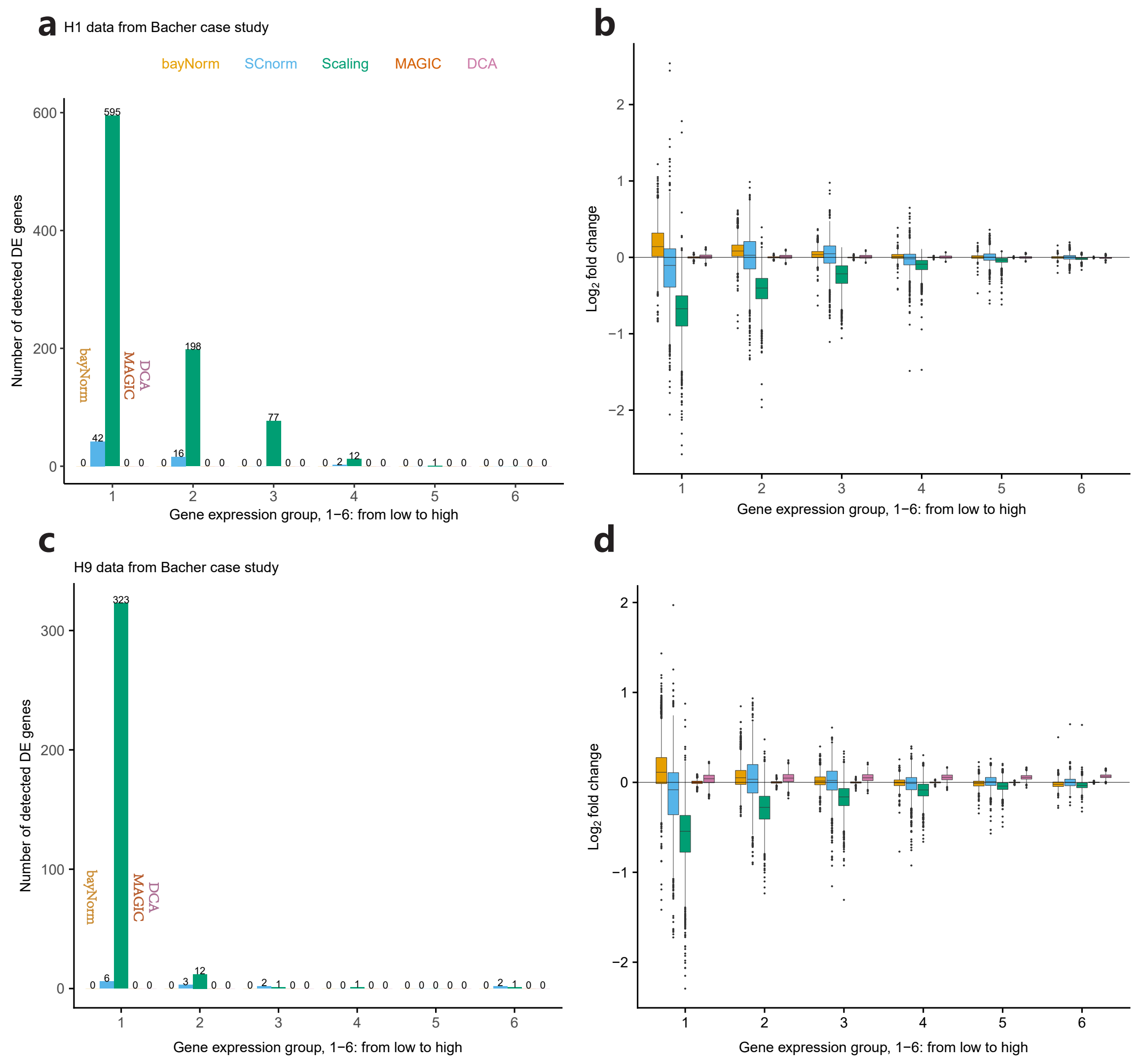

Figure S13: bayNorm correction for differences in sequencing depths. Data from H1 (a-b, 13181 genes in total) and H9 (c-d, 13195 genes in total) hESC cells are shown. (a) Number of DE genes called by MAST as a function of gene expression groups ($P_{\mathrm{MAST}} < 0.05$). (b) Log_2 fold change as a function of gene expression group. In (a) bayNorm is based on 20 posterior samples (3D array). In (b), bayNorm is based on the mean of posteriors (2D array).

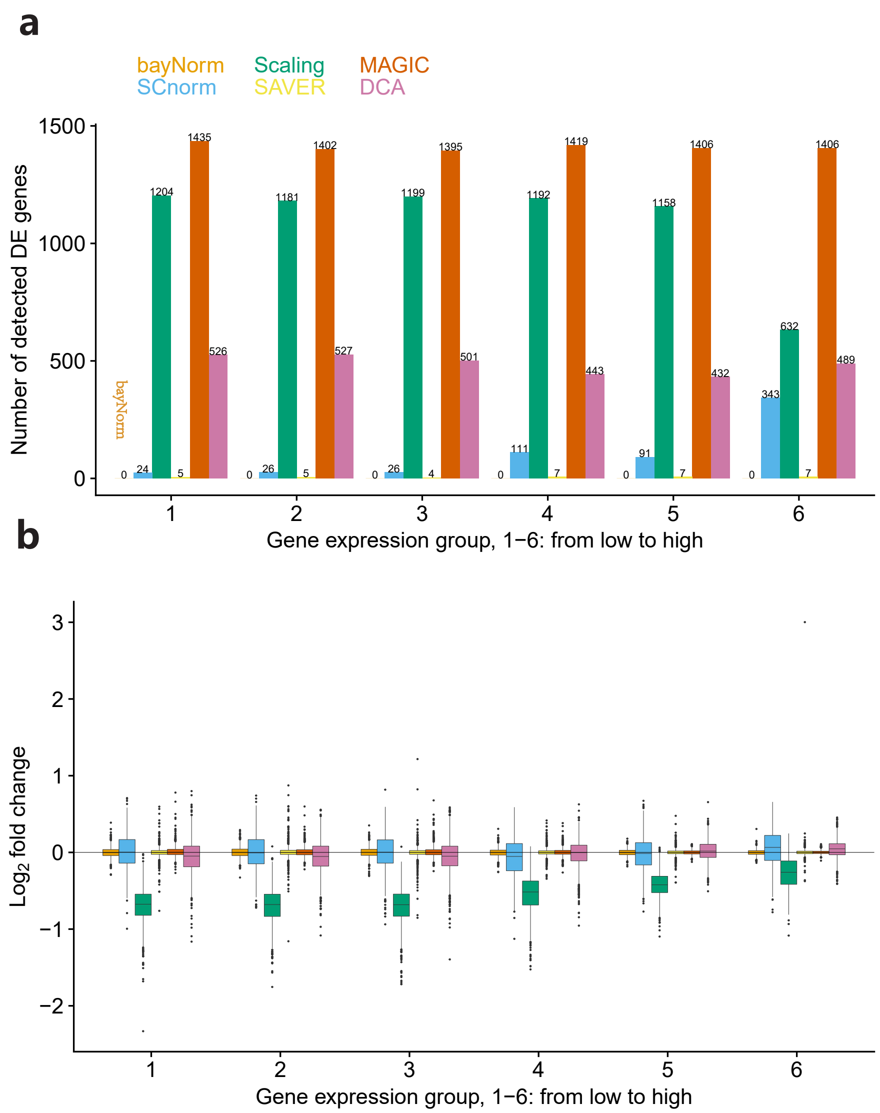

Figure S14: Simulation analysis, SIM noDE study I (see Supplementary Information for details about simulation studies). (a) Number of detected DE genes (MAST) as a function of expression group ($P_{\text{MAST}} < 0.05$, 9999 genes in total). (b) Log_2 fold change of mean expression between two groups for different expression groups. For bayNorm and SAVER, 10 samples were generated and the median of p-values across the 10 samples was used in (a). In (b), bayNorm and SAVER are based on mean of posterior distributions.

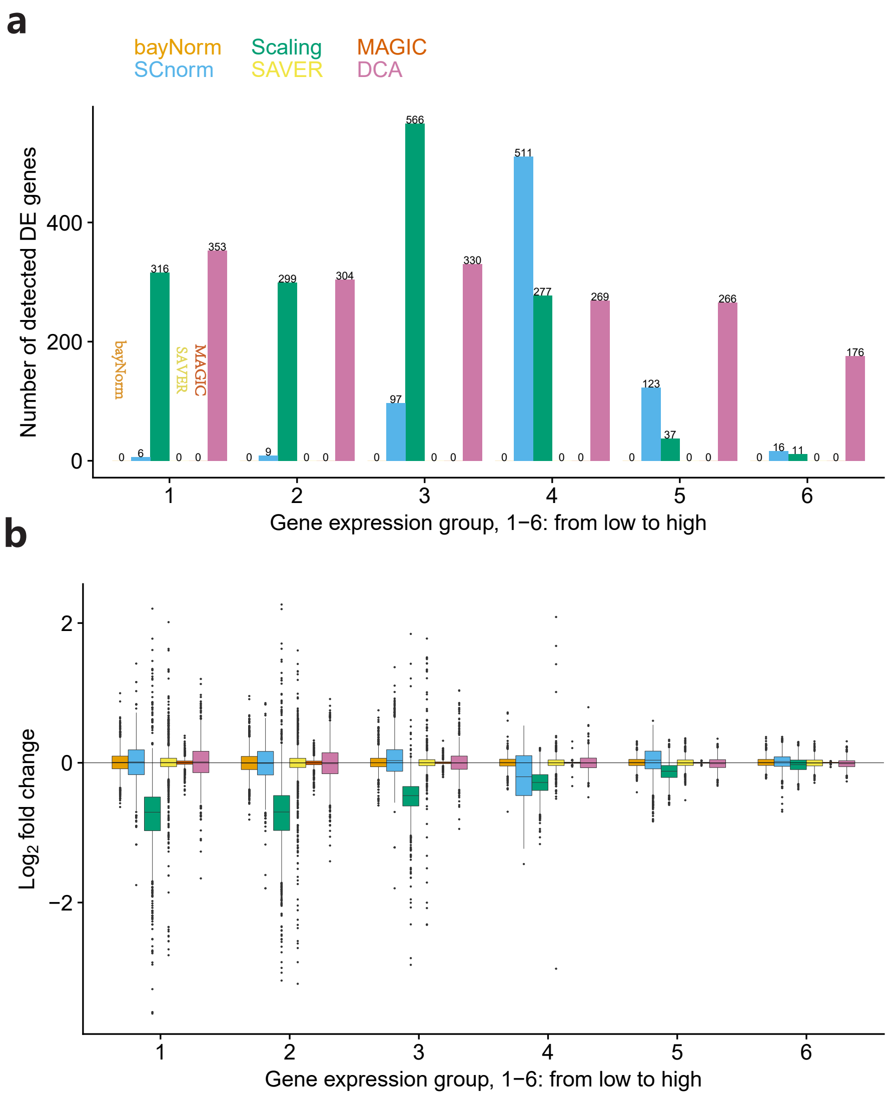

Figure S15: Simulation analysis, SIM noDE study II (see Supplementary Information for details about simulation studies). (a) Number of detected DE genes (MAST) as a function of expression group ($P_{\text{MAST}} <$ 0.05, 9598 genes in total). (b) Log_2 fold change of mean expression between two groups for different expression groups. For bayNorm and SAVER, 10 samples were generated and the median of p-values across the 10 samples was used in (a). In (b), bayNorm and SAVER are based on mean of posterior distributions.

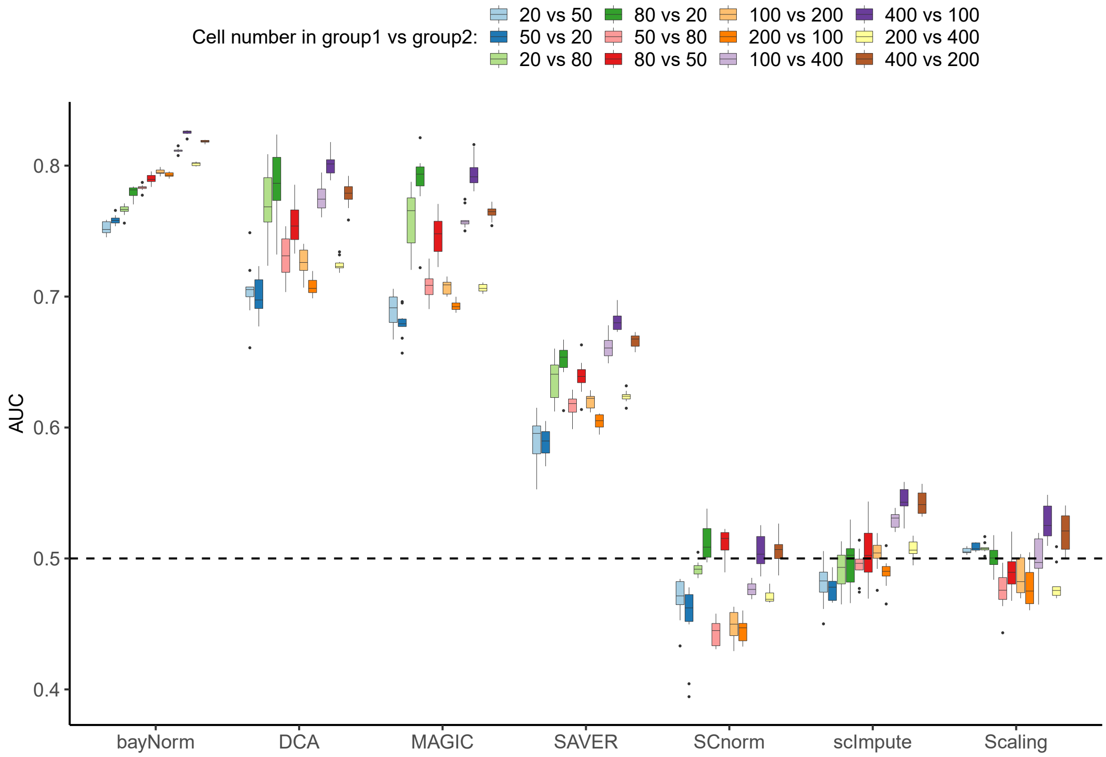

Figure S16: DE detection for unbalanced groups of cells (UMI data from the Soumillon study). Ten samples of 20, 50, 80, 100, 200 and 400 cells were randomly selected from each group. DE detection was performed using MAST between groups as described at the top of the figure using a list of DE genes obtained from matched bulk RNA-seq data as a benchmark (1000 genes with the largest magnitude of log fold-change between the D3T0 and D3T7 samples, [18]). For bayNorm and SAVER, 3D arrays were used.

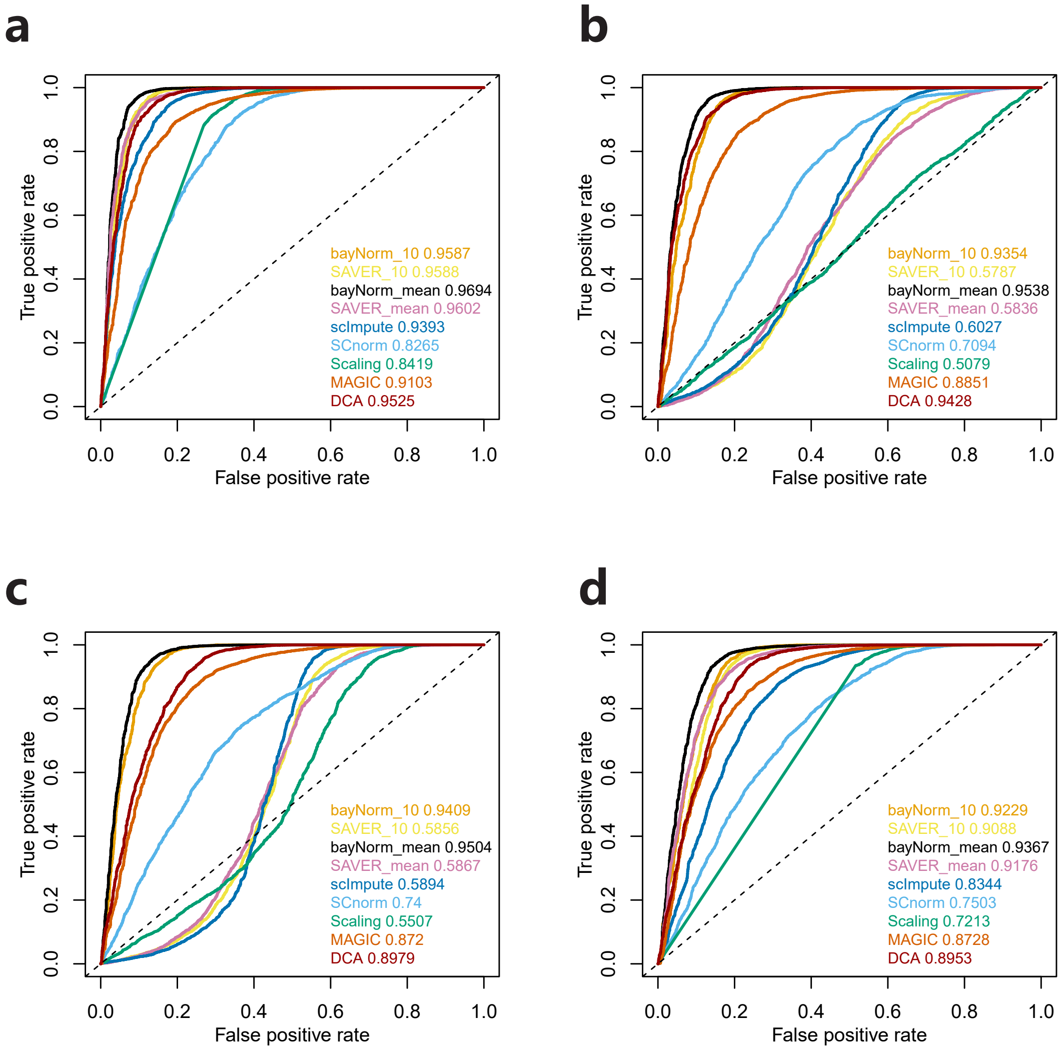

Figure S17: DE analysis on simulated scRNA-seq data, SIM DE study (see Supplementary Information for details about simulation studies). (a-d) represent four simulation scenarios and DE detection is based on MAST. (a) SIM I: mean capture efficiencies are set to 0.1 for the two groups. (b) SIM II: mean capture efficiencies are set to 0.05 and 0.1 in group 1 and group 2 respectively. (c) SIM III: mean capture efficiencies are set to 0.1 and 0.05 in group 1 and group 2 respectively. (d) SIM IV: mean capture efficiencies are set to 0.05 in both groups. 2000 out of 10000 genes were simulated to be DE genes in group 1. bayNorm_10 and SAVER_10 are based on 10 samples from posterior distributions (3D arrays). DE detection was performed on each sample and the median of adjusted MAST P-values were used.

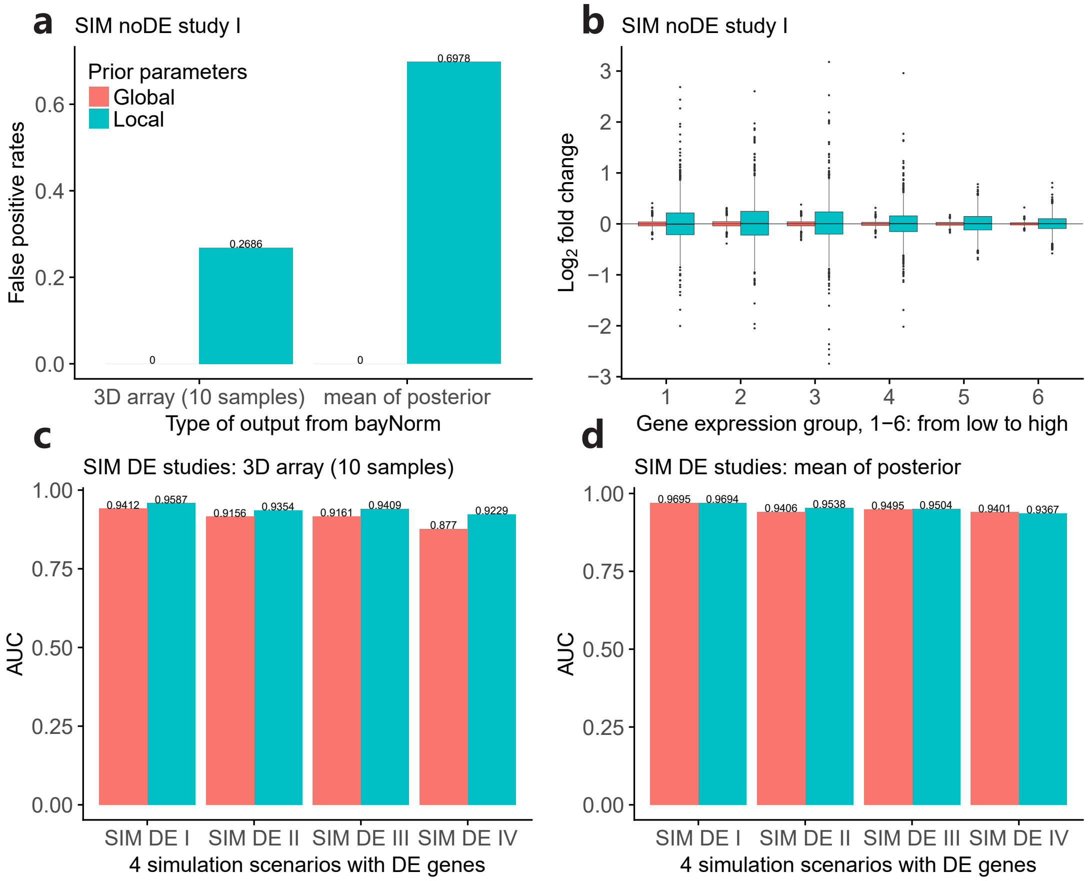

Figure S18: Impact of global or local priors on the DE detection for simulated samples with different sequencing depths. (a-b) Simulation analysis, SIM noDE study I, with no DE genes. (c-d) SIM DE studies I-IV where 2000 out of 10000 genes were simulated to be differentially expressed in the first group. (c) and (d) are based on 10 samples (3D array) and mean (2D array) output from bayNorm respectively.

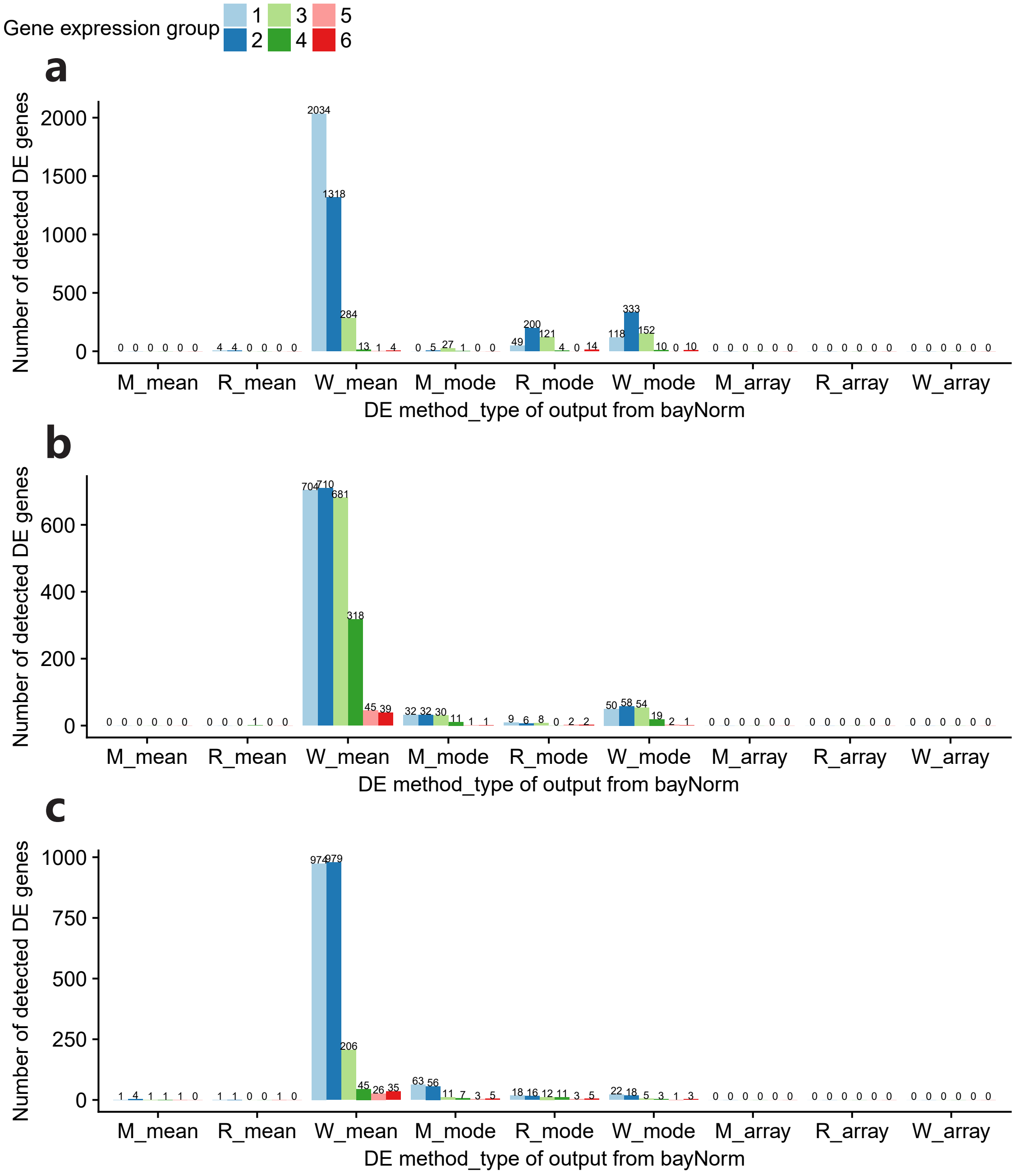

Figure S19: Impact of different DE methods and different types of output from bayNorm on samples with different sequencing depths. M stands for MAST, R stands for ROTS and W stands for Wilcoxon test. Mean (2D array), mode (2D array) and array (3D array) stands for the three different types of output from bayNorm (see Fig S1). For the 3D array output from bayNorm, each DE method was applied on each one of 10 samples from the posterior distribution, and the median of P-values was used. (a) H1 hESC data from the Bacher study. (b) Simulated data, SIM noDE study I. (c) Simulated data, SIM noDE study II (See Supplementary Information for details about simulation studies). Genes were categorized into 6 groups according to their mean expression (1-low – 6-high).

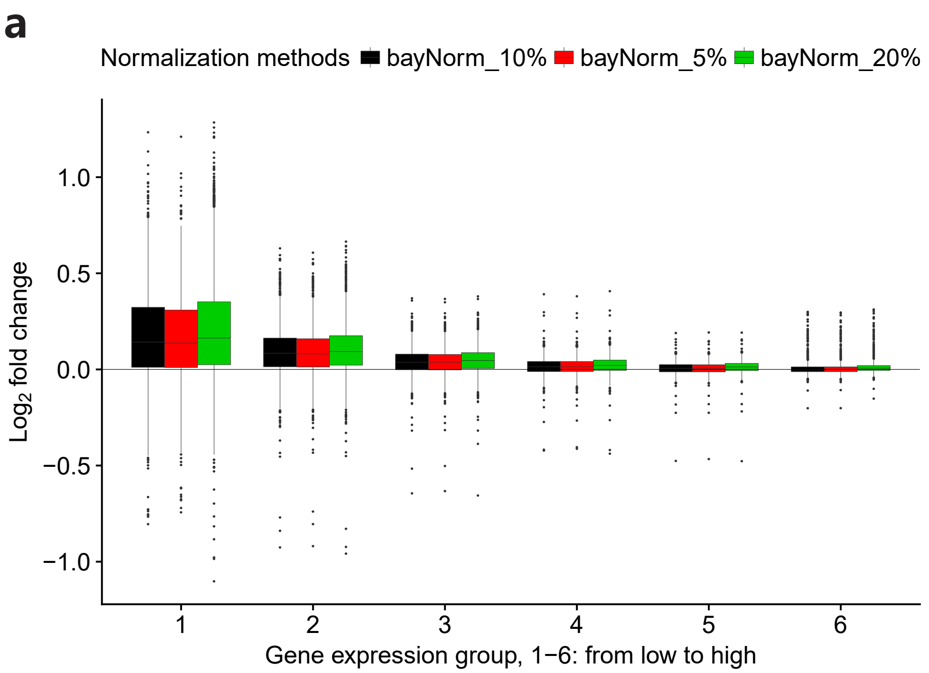

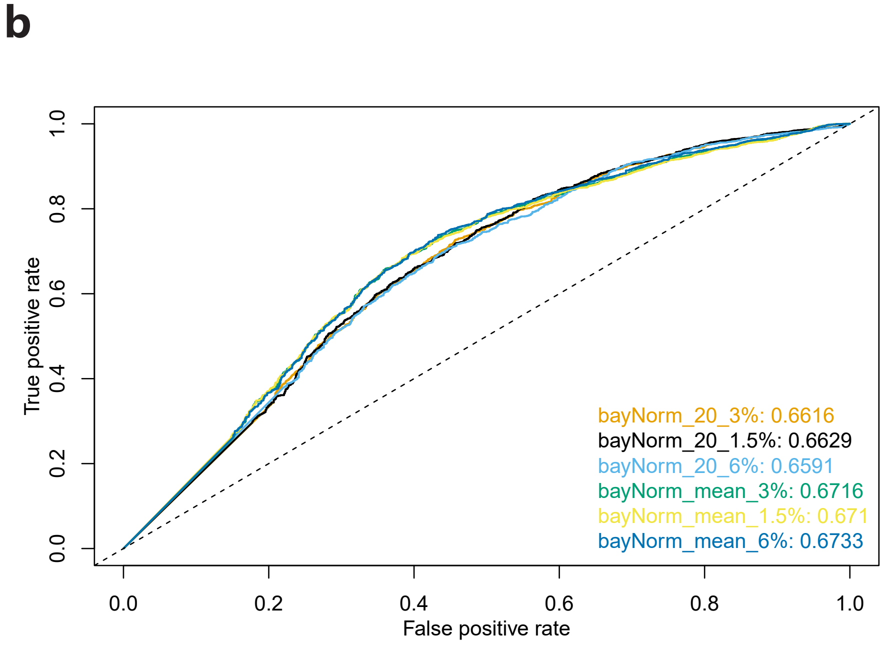

Figure S20: Impact of different mean capture efficiencies on DE analysis based on Bacher and Islam studies. (a) For data from the Bacher study, mean capture efficiencies were set to 5%, 10% or 20%. Results are based on mean of posterior output (2D array) from bayNorm. The result of DE detection was not shown as no genes were called DE at threshold 0.05. (b) Islam study. Mean capture efficiencies were set to 1.5%, 3% and 6%. mean (2D array) or 20 samples (3D array) were used for DE detection.

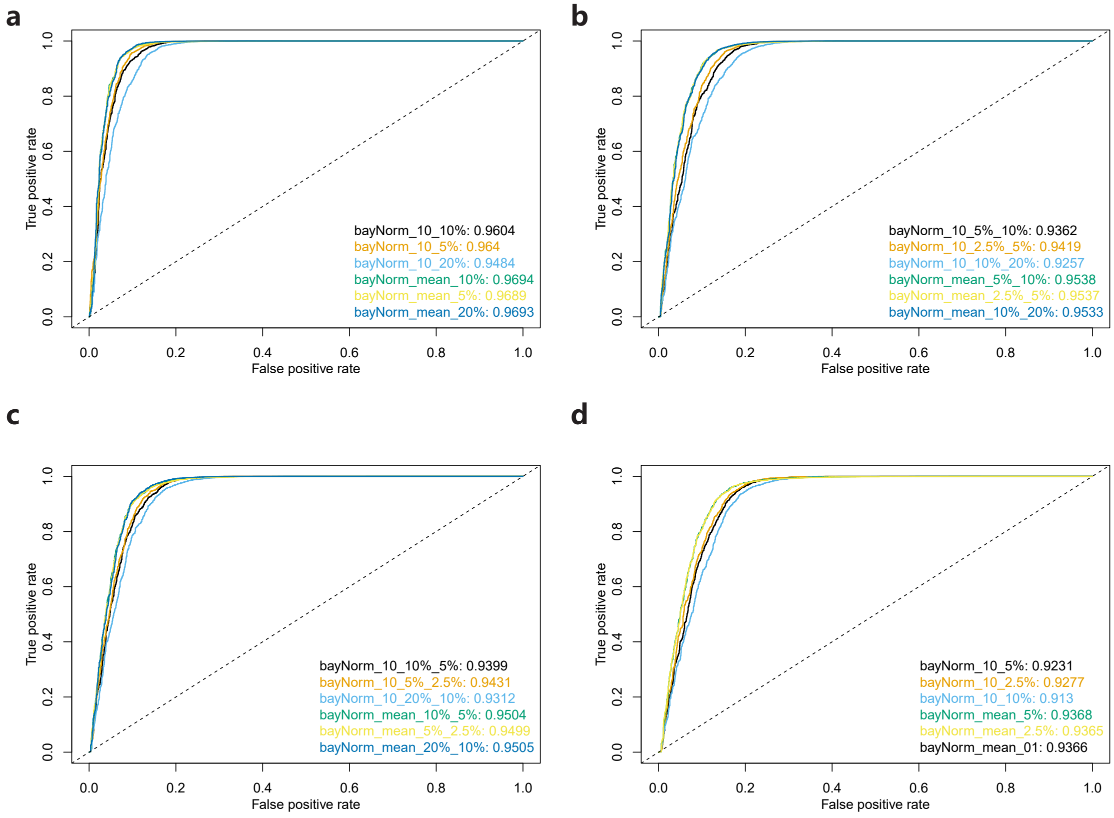

Figure S21: Impact of different mean capture efficiencies on DE detection in simulated studies (see Supplementary Information for details about simulation studies). (a) SIM I from our SIM DE study. Mean capture efficiencies were set to 5%, 10% or 20% using either mean of posterior (2D array) or 10 samples generated from the posterior distributions (3D array) as normalized data. (b) SIM II from our SIM DE study. Mean capture efficiencies were set to twice or half of the original magnitude. (c) SIM III from our SIM DE study, Mean capture efficiencies were set to twice or half of the original magnitude. (d) SIM IV from our SIM DE study. Mean capture efficiencies were set to 2.5%, 5% or 10%. "mean" stands for the mean version's output from bayNorm (2D array). Otherwise the number indicates the number of samples generated from posterior distribution (3D array). DE was performed on each sample, the median of MAST P-values were used.

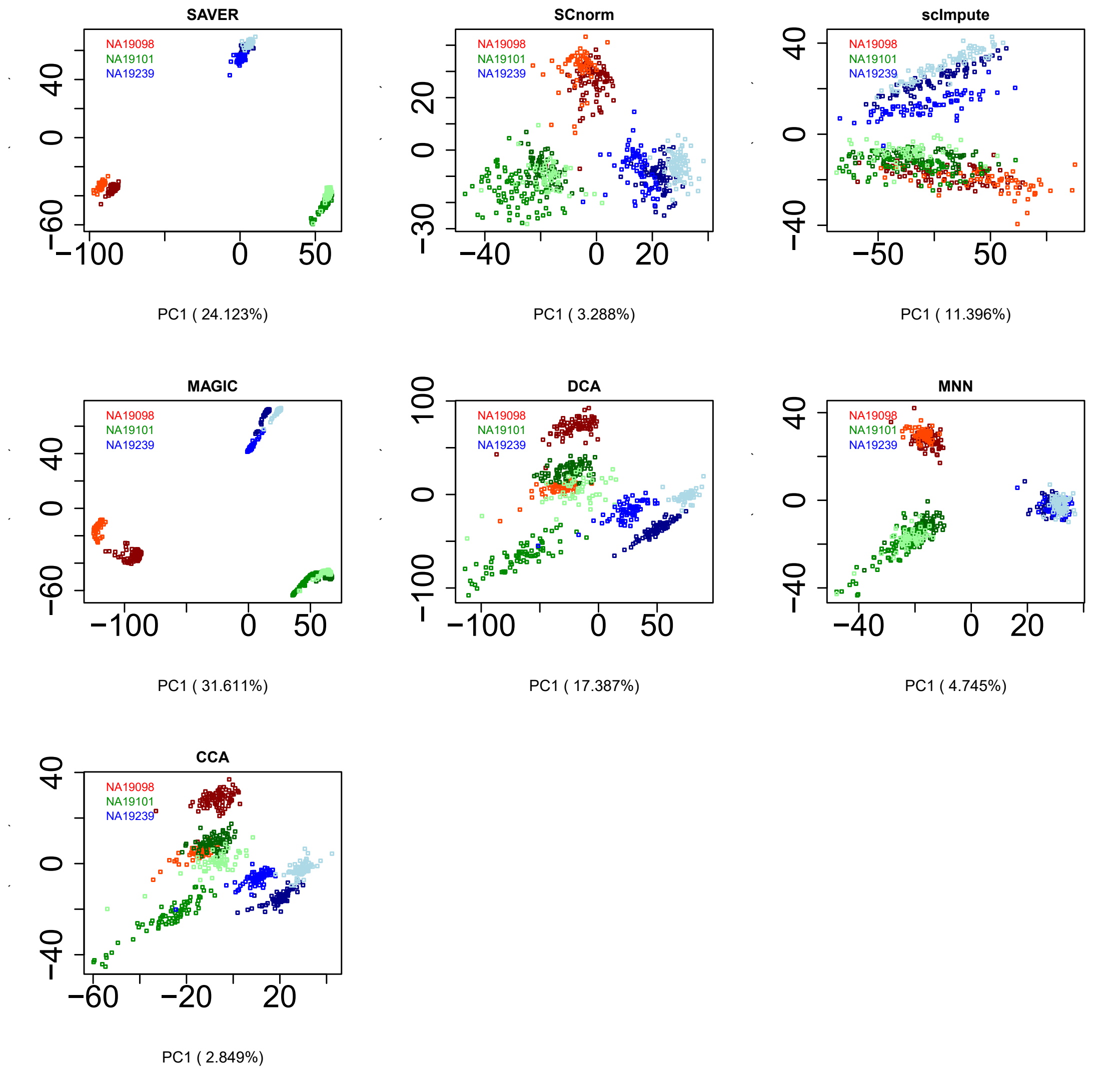

Figure S22: PCA plots of scRNA-seq data normalised using different methods (Tung study). PCA plot of SAVER normalized data is based on the mean version's output (2D array). Different colours represent different individuals. Different shades of the same colour stands for a specific batch within each individual.

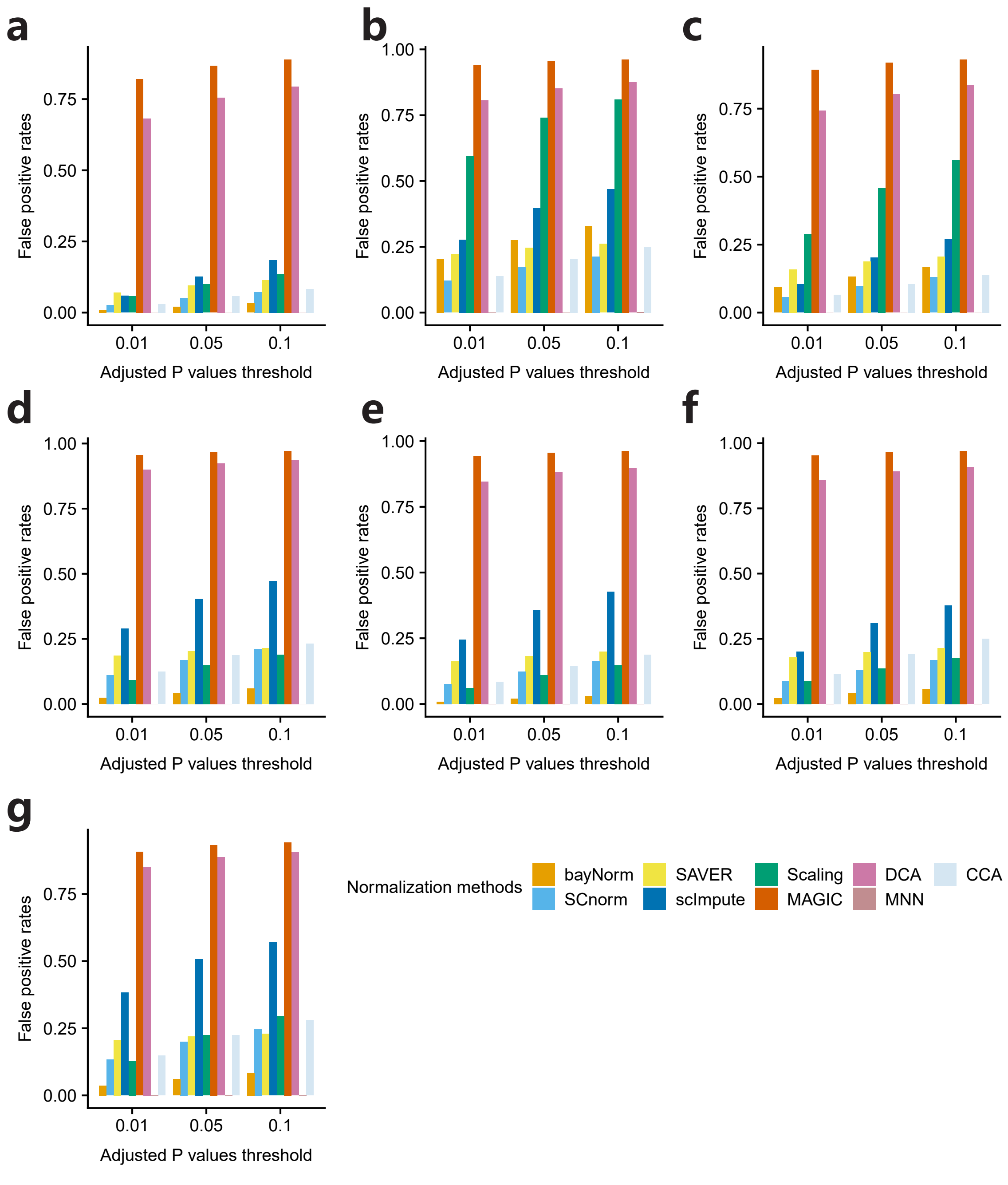

Figure S23: DE detection between scRNA-seq data for different batches within single individuals. (a-c) Individual NA19101, (d-f) Individual NA19239, (g) Individual NA19098. (a), (d) and (g) show DE detection between batch 1 and batch 3 (batch 2 was not considered as suggested in the Tung study). (b) and (e) show DE detection between batch 1 and batch 2. (c) and (f) show DE detection between batch 2 and batch 3. Results of bayNorm and SAVER are based on 5 samples from posterior distributions (3D array).

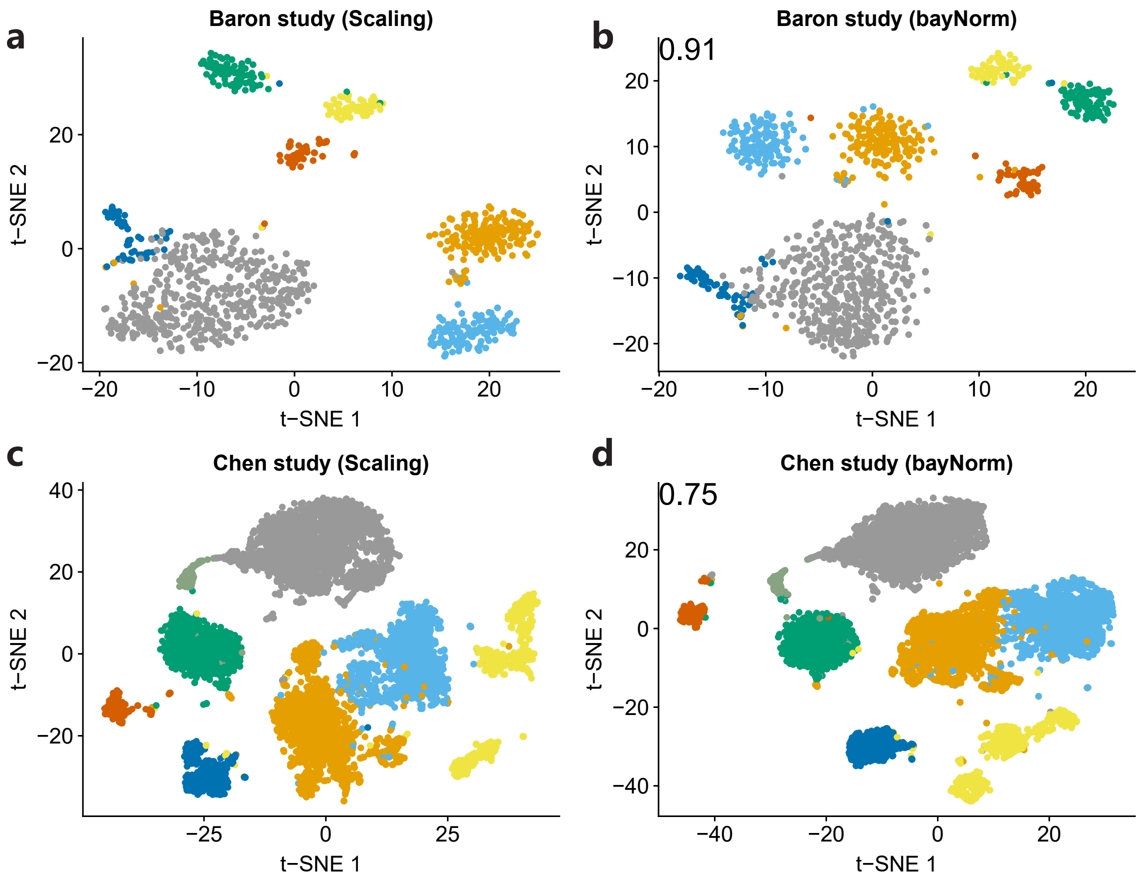

Figure S24: Using Scaling normalized data as baseline, the Jaccard was computed using cell lablels found in both Scaling and bayNorm normalized data.

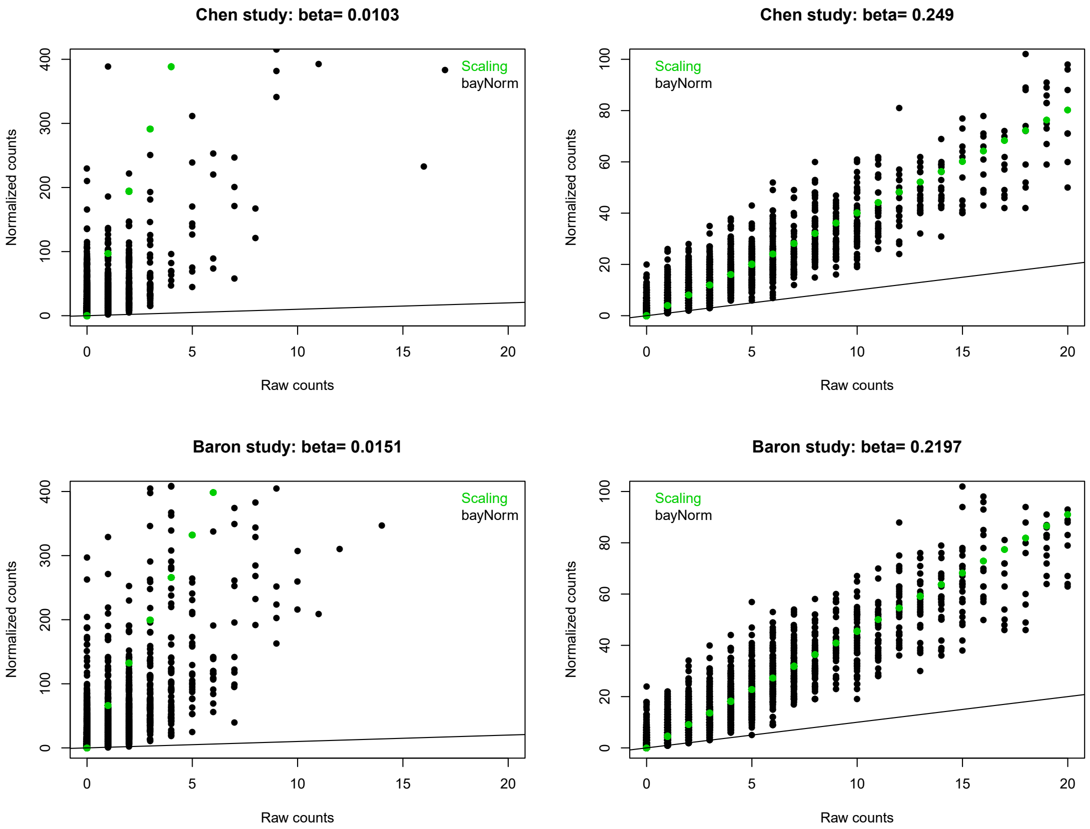

Figure S25: Effect of bayNorm on low counts (compared with Scaling method, straight line is y = x). The first and second columns represent cells with lowest and highest capture efficiency respectively in each study.

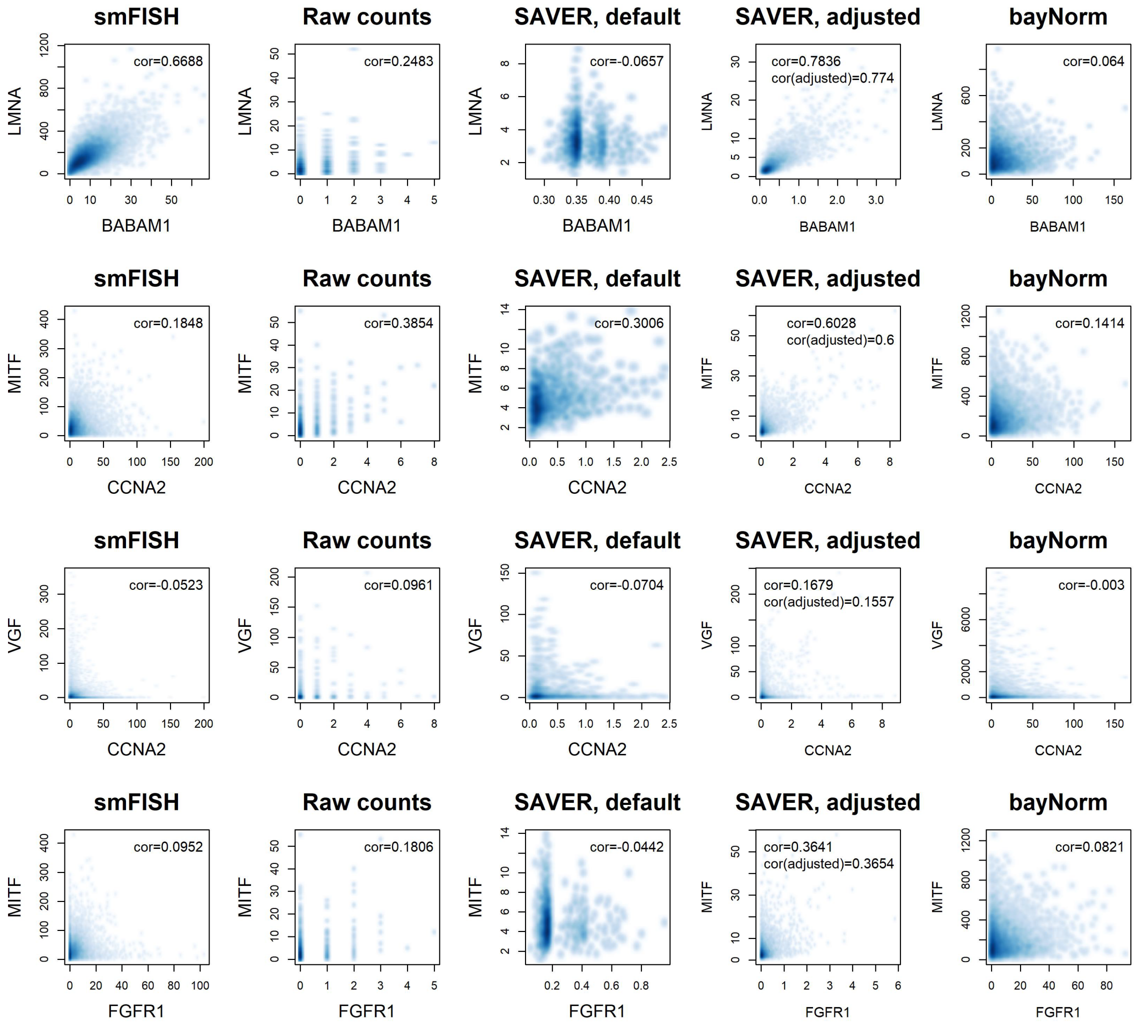

Figure S26: Gene-gene correlation in Torre study.

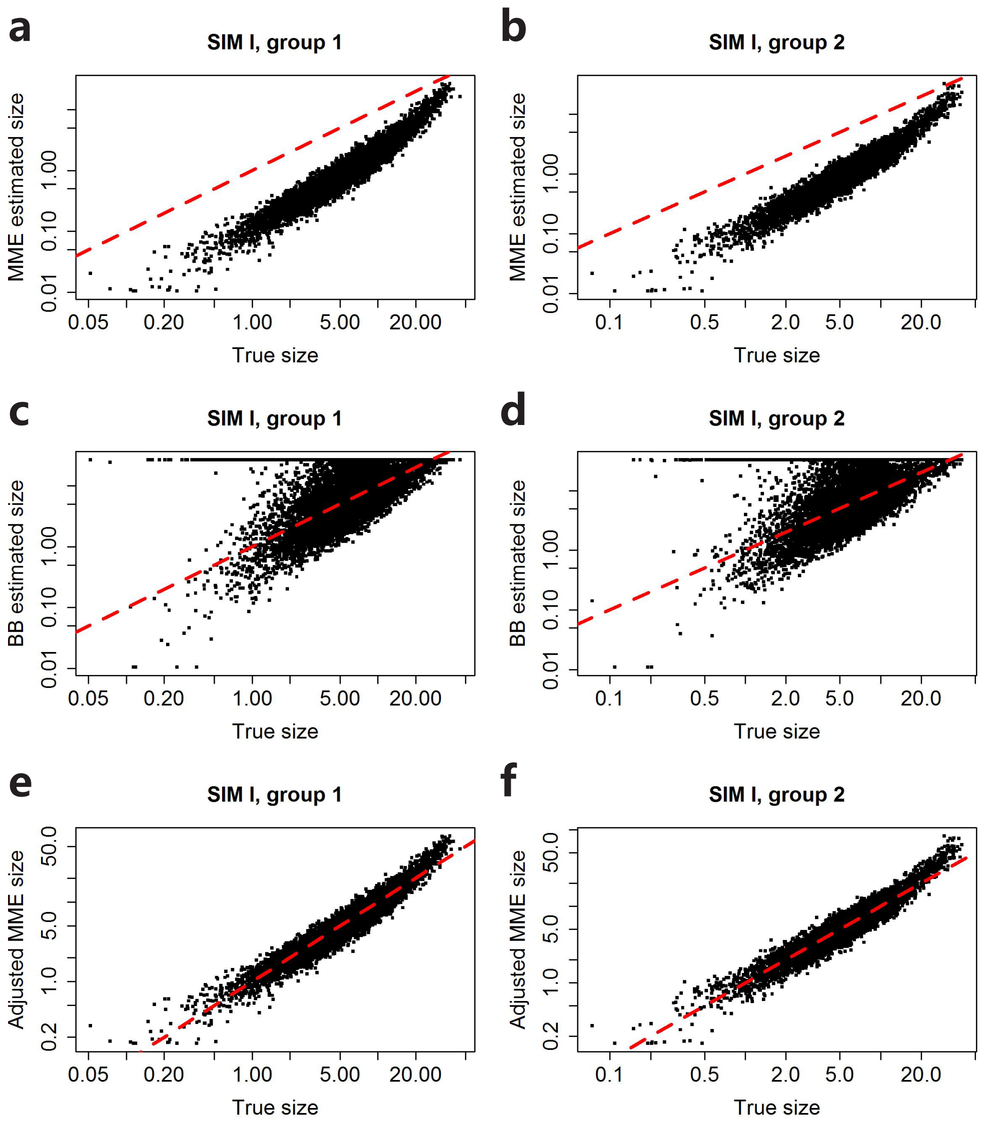

Figure S27: Estimation of the size factor (dispersion parameter) of the negative binomial prior distribution based on simulation studies (see Supplementary Information for details about simulation studies). (a-b) comparison between the MME estimated size and the true size. (c-d) comparison between the BB estimated size and the true size. (e-f) comparison between the adjusted MME size and the true size. 2000 out of 10000 genes were simulated to be differentially expressed in group 1. Results are similar for other three simulated datasets (SIM DE II-IV).

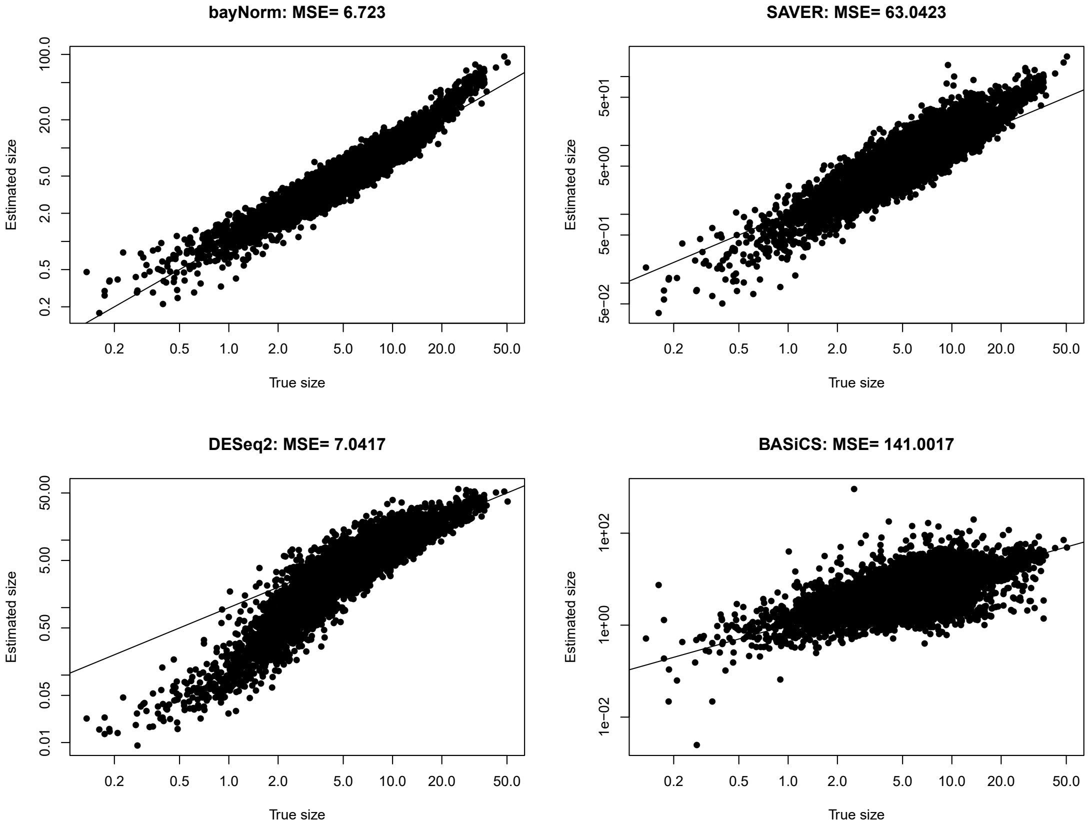

Figure S28: bayNorm results in better estimation of the dispersion parameter of negative binomial distribution (ϕ) compared to SAVER, DESeq2, BASiCS . Result is based on the group 2 of SIM DE study II (see Supplementary Note 2 and 3. For BASiCS, we applied it without spike-ins, see [27].
